# Visualization of Traditional Chinese Medicine Formulas: Development and Usability Study

**DOI:** 10.2196/40805

**Published:** 2023-04-21

**Authors:** Zhiyue Wu, Suyuan Peng, Liang Zhou

**Affiliations:** 1 Institute of Medical Technology Peking University Beijing China; 2 National Institute of Health Data Science Peking University Beijing China

**Keywords:** visualization, Chinese medicine formulas, interactive data analysis, traditional Chinese medicine, multifaceted data visualization, five elements

## Abstract

**Background:**

Traditional Chinese medicine (TCM) formulas are combinations of Chinese herbal medicines. Knowledge of classic medicine formulas is the basis of TCM diagnosis and treatment and is the core of TCM inheritance. The large number and flexibility of medicine formulas make memorization difficult, and understanding their composition rules is even more difficult. The multifaceted and multidimensional properties of herbal medicines are important for understanding the formula; however, these are usually separated from the formula information. Furthermore, these data are presented as text and cannot be analyzed jointly and interactively.

**Objective:**

We aimed to devise a visualization method for TCM formulas that shows the composition of medicine formulas and the multidimensional properties of herbal medicines involved and supports the comparison of medicine formulas.

**Methods:**

A TCM formula visualization method with multiple linked views is proposed and implemented as a web-based tool after close collaboration between visualization and TCM experts. The composition of medicine formulas is visualized in a formula view with a similarity-based layout supporting the comparison of compositing herbs; a shared herb view complements the formula view by showing all overlaps of pair-wise formulas; and a dimensionality-reduction plot of herbs enables the visualization of multidimensional herb properties. The usefulness of the tool was evaluated through a usability study with TCM experts.

**Results:**

Our method was applied to 2 typical categories of medicine formulas, namely tonic formulas and heat-clearing formulas, which contain 20 and 26 formulas composed of 58 and 73 herbal medicines, respectively. Each herbal medicine has a 23-dimensional characterizing attribute. In the usability study, TCM experts explored the 2 data sets with our web-based tool and quickly gained insight into formulas and herbs of interest, as well as the overall features of the formula groups that are difficult to identify with the traditional text-based method. Moreover, feedback from the experts indicated the usefulness of the proposed method.

**Conclusions:**

Our TCM formula visualization method is able to visualize and compare complex medicine formulas and the multidimensional attributes of herbal medicines using a web-based tool. TCM experts gained insights into 2 typical medicine formula categories using our method. Overall, the new method is a promising first step toward new TCM formula education and analysis methodologies.

## Introduction

Understanding and applying classical medicine formulas is the basis of traditional Chinese medicine (TCM) diagnosis and treatment and is the core of TCM inheritance. We use the term medicine formulas and herbal formulas interchangeably. Syndrome differentiation and treatment is a core method used in TCM. In clinical practice, prescriptions are based on classical medicine formulas, and the corresponding medicines may be adjusted according to the symptoms of patients. A typical prescription may contain several medicine formulas, but it is a challenge to identify the involved formulas and understand their effects.

Learning and teaching formulas for Chinese medicine is difficult. Traditional education methods involve reciting classical medicine formulas based on their composition rules [1,2]. However, formula information is presented in text (Table 1) or static figures and pictures [3], and the composition rules could not be intuitively understood. Data mining and some visual presentations are adopted in the existing computerized analysis of TCM formulas [4-6]. However, these methods are query based and do not allow users to interactively explore medicine formulas, and the relatively simple visualization cannot provide an overview of a group of medicine formulas or an in-depth comparison of formulas.

In this study, we propose a visualization method for TCM formulas to assist in the learning of the subject. Our method provides an overview of a set of formulas and their compositing medicines and an interactive exploration of the association between formulas and herbs. The usefulness of our method was demonstrated using 2 use cases of typical medicine formula groups in a usability study.

The target audience of our method was medical students learning TCM formulas. However, TCM doctors and patients could also benefit from our method to better understand the formulas or prescriptions.

In this paper, Pinyin—the standard romanization system of Chinese—is used for the names of formulas and medicines, and the corresponding Chinese characters are provided in parentheses. A conversion table for Pinyin, Chinese characters, English, and Latin is provided in Multimedia Appendix 1. High resolution figures can be found in Multimedia Appendix 2.

**Table 1:** Part of the original text–based medicine formula information summarized from the textbook Chinese Herbal Formulas (Tenth Edition) [7].

Formula	Medicines
*Bazhentang*^a^ (八珍汤)	Renshen (Ginseng, 人参)^b.c^ Shudihuang (Prepared Rehmannia Root, 熟地黄)^c^ Danggui (root of Chinese Angelica, 当归) Chuanxiong (Chuanxiong Rhizoma, 川芎) Baizhu (rhizome of Largehead Atractylodes, 白术) Fuling (Indian Bread, 茯苓) Baishao (White peony root, 白芍) Zhigancao (liquorice root, 炙甘草) Shengjiang (Fresh Ginger, 生姜) Dazao (Jujube Chinese date, 大枣)
*Shenlingbaizhusan* (参苓白术散)	Renshen (Ginseng, 人参)^c^ Baizhu (rhizome of Largehead Atractylodes, 白术)^c^ Fuling (Indian Bread, 茯苓)^c^ Lianzi (Lotus Seed, 莲子) Yiyiren (seed of Jobstears, 薏苡仁) Shanyao (Common Yam Rhizome, 山药) Jiegeng (Platycodon Root, 桔梗) Dazao (Jujube Chinese date, 大枣) Gancao (root of Ural Licorice, 甘草) Sharen (Villous Amomum Fruit, 砂仁) Baibiandou (White Hyacinth Bean, 白扁豆)
*Shengmaisan* (生脉散)	Renshen (Ginseng, 人参)^c^ Maidong (Dwarf lilyturf tuber, 麦冬) Wuweizi (Schisandrae Chinensis Fructus, 五味子)
*Sijunzitang* (四君子汤)	Renshen (Ginseng, 人参)^c^ Gancao (root of Ural Licorice, 甘草) Baizhu (rhizome of Largehead Atractylodes, 白术) Fuling (Indian Bread, 茯苓)
*Dabuyinwan* (大补阴丸)	Shudihuang (Prepared Rehmannia Root, 熟地黄)^c^ Guijia (Tortose's Carapae and Plastron, 龟甲)^c^ Huangbo (Phellodendron bark, 黄柏) Zhimu (rhizome of Common Amarrhe, 知母)
*Siwutang* (四物汤)	Shudihuang (Prepared Rehmannia Root, 熟地黄)^c^ Baishao (White peony root, 白芍) Chuanxiong (Chuanxiong Rhizoma, 川芎) Danggui (root of Chinese Angelica, 当归)
*Dihuangyinzi* (地黄饮子)	Shudihuang (Prepared Rehmannia Root, 熟地黄)^c^ Shanzhuyu (Asiatic Cornelian Cherry Fruit, 山茱萸)^c^ Roucongrong (Desertliving Cistanche, 肉苁蓉)^c^ Bajitian (Morindae Officilis Radix, 巴戟天)^c^ Maidong (Dwarf lilyturf tuber, 麦冬) Yuanzhi (Thinleaf Milkwort Root, 远志) Shengjiang (Fresh Ginger, 生姜) Fuzi (Common Monkshood Daughter Root, 附子) Fuling (Indian Bread, 茯苓) Dazao (Jujube Chinese date, 大枣) Wuweizi (Schisandrae Chinensis Fructus, 五味子) Shihu (Noble Dendrobium Stem Herb, 石斛) Shichangpu (Grassleaf Sweetflag Rhizome, 石菖蒲) Rougui (Cassia Bark, 肉桂) Bohe (Mentha, Peppermint, 薄荷)

^a^The italicization represents the Pinyin name of formulas.

^b^Pinyin (English name, Chinese name).

^c^Principal herb or herbs.

## Methods

### Data Descriptions

Classifications of Chinese herbal medicines are multifaceted and multileveled [2]. Siqi (四气), Wuwei (五味), and Guijing (归经) are the basic attributes for herb classification and have been an important part of TCM research. Siqi represents the properties of Chinese herbal medicines according to their functions on the human body: cold (寒), hot (热), warm (温), and cool (凉). In addition, herbs with gentle properties are namely calm (平). Wuwei means flavors: pungent (辛), sweet (甘), sour (酸), bitter (苦), salty (咸), tasteless (淡), and astringent (涩). It is believed that these factors are associated with body heat production processes or metabolic activities and may also play a role in the digestive system, nervous system, and cardiovascular system [8]. Guijing regards the orientation of Chinese herbal medicines, which is to closely connect the functions of herbs with the organs and meridians (脏腑经络) of the human body.

Another important concept for herbs in the formula is Jun-Chen-Zuo-Shi (君臣佐使). Jun-Chen-Zuo-Shi is the principle of the compatibility of TCM formulas. Junyao (君药), namely, principal herbs as used hereafter, plays a major role against the main disease or syndrome. It is the primary herb used in the formulas. Footnote c in Table 1 indicates Junyao in the corresponding formulas.

In this work, the medicine formulas data were extracted from the key medicine formulas of the textbook *Chinese Herbal Formulas (Tenth Edition)* [7], as shown in Table 1. Multidimensional herb attribute data were retrieved from the SymMap database [9]. Siqi has 5 dimensions: cold, hot, warm, cool, and calm. Wuwei has 7 dimensions: pungent, sweet, sour, bitter, salty, tasteless, and astringent. Guijing has 11 orientations: liver meridian, heart meridian, spleen meridian, lung meridian, kidney meridian, bladder meridian, large intestine meridian, small intestine meridian, stomach meridian , gallbladder meridian, and pericardium meridian. These properties were combined and represented as a 23-dimensional vector for each herb.

### Ethical Considerations

This study did not involve human subjects research. The data used in this study were obtained from a publicly available database and a textbook.

### Requirement Analysis and Method Overview

Our goal was to devise a joint visualization method of medicine formulas and the attributes of corresponding herbs. The visual design should support the comparison of formulas and facilitate the classification of herbs based on their properties (Siqi, Wuwei, and Guijing). Visualization and TCM experts worked closely together to analyze the requirements of the visual analysis method for medicine formulas. The requirements are summarized as follows:

- Requirement 1: clear visualization of medicine formulas
- Requirement 2: comparing different medicine formulas with ease
- Requirement 3: principal herbs should be highlighted
- Requirement 4: associating medicine formulas and attributes of the corresponding herbs
- Requirement 5: visual elements should be effectively perceived
- Requirement 6: interactions should be easy
- Requirement 7: visual designs should reflect general concepts of TCM

Our method is the result of an iterative development process using quick prototypes. Prototypes were realized based on the requirements and proposed to the TCM expert (SP, one of the authors), and improvements were made based on the feedback of the TCM expert.

**Figure 1:**
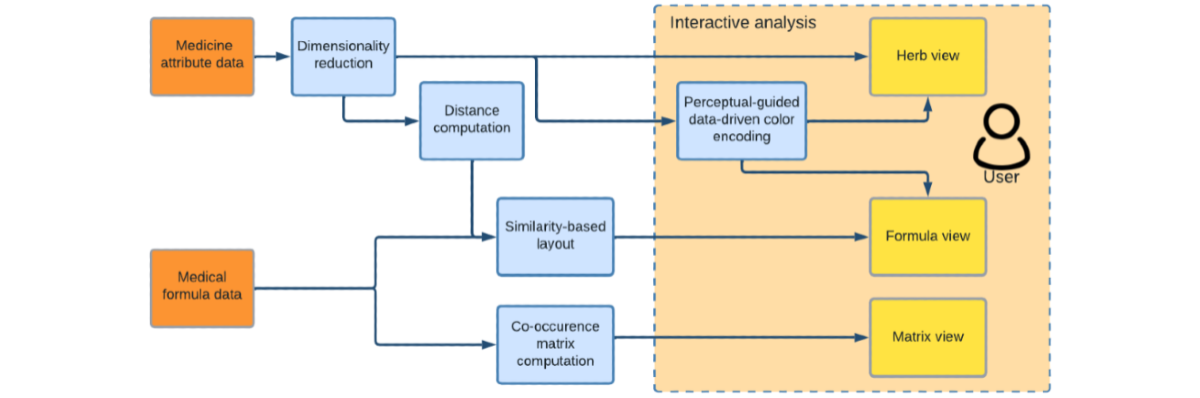
The workflow of our method.

The workflow of our method is shown in Figure 1: the medicine formulas information and the multidimensional medicine attribute data are prepared as the input; medicine attribute data are projected to the low-dimensional space (2D) and pair-wise distances are calculated; medicine formulas data are arranged by our similarity-based layout algorithm and visualized as an icicle plot; shared herbs of each pair of formulas are calculated and visualized as a matrix; and next, colors are designed for herbs using our perceptual-guided, data-driven color-encoding method.

### Dimensionality Reduction and Distance Computation

The attributes of an herbal medicine can be written as an M-dimensional (M=23) vector **P** of binary valued elements:



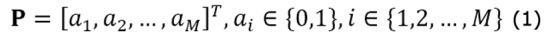



The M-dimensional space is then dimensionality reduced to 2D with a vector **p** of real values:



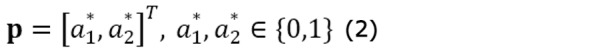



Uniform manifold approximation and projection for dimension reduction (UMAP) [10] is used for its structure preservation ability and computational efficiency. The distance between the herbs is the basis of our subsequent similarity-based layout computation and visualization. We defined the distance *d (u, v)* between 2 herbs *u* and v as the L2-norm, that is, Euclidean distance, between their corresponding 2D vectors **p***_u_* and **p***_v_*, respectively:

*d (u, v)* = ||**p**_u_ – **p**_v_||. **(3)**

The distance between **P**_u_ and **P**_v_ in the original M-dimensional space is also considered. However, our experiment shows that the difficulty of discriminating herbs based on the distance with **P** is higher than that with the projected vectors **p**, and the resulting visualization based on **P** is more difficult to compare and comes with more visual clutter.

### Formulas Visualization

#### Domain Expert Evaluation of Set Visualization Methods

Typically, a dozen formulas and even more herbs are included in a category of formulas. From a set visualization perspective, both the number of sets and set elements are large; therefore, a suitable visualization that scales well and is easily understandable is required.

We evaluated popular set visualization techniques to design a proper set visualization method using a TCM expert (SP). The figures of an Euler diagram, a node-link diagram, and matrix-based methods included in a set visualization survey paper [11] were shown to the TCM expert. The expert was asked to rank the feasibility of these methods for medicine formulas visualization based on the scalability, the ease of understanding, and the support for comparison. The matrix-based method was ranked first by the TCM expert, followed by the node-link diagram, the Euler diagram, and the overlay.

On the basis for this informal evaluation, we decided to devise a sparse matrix-based method based on the evaluation to show formulas and corresponding medicines to meet requirements 1 and 2. To support the analysis of overlapping herbs within formulas, a co-occurrence matrix view is used to complement the formula view.

#### Icicle Plot of Medicine Formulas

Our formula-medicine matrix (set-element matrix) treats formulas (sets) as columns and herbs (elements) as rows. The matrix can be shown with a sparse representation as a collection of formula columns of their corresponding herb rows. This representation is similar to that of an icicle plot for hierarchical visualization. It has the potential to support the comparison of similar medicine formulas if properly laid out. Furthermore, the icicle plot allows for the encoding of herbs in a hierarchy to separate the principal herbs from other herbs.

**Figure 2:**
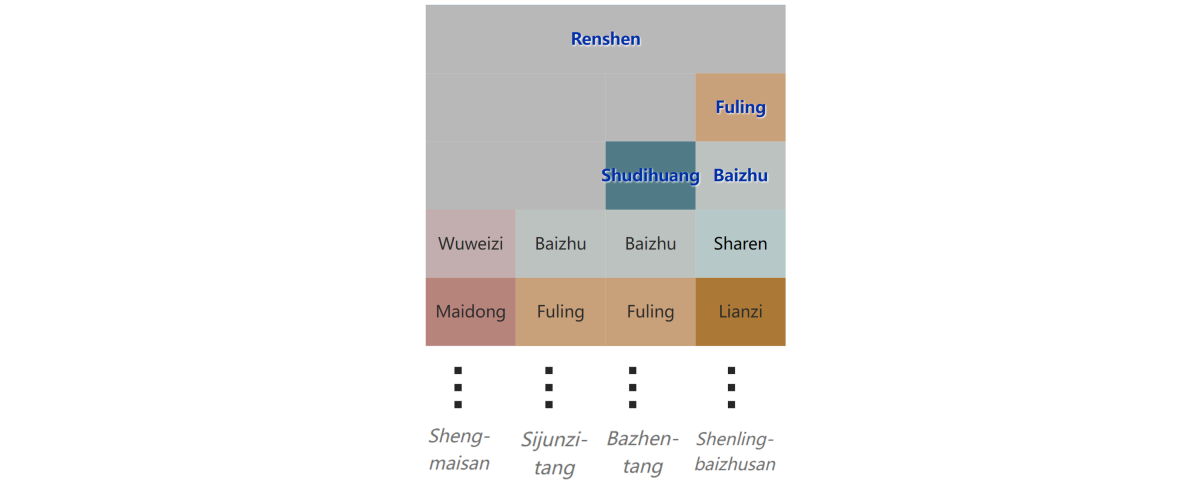
The design of the icicle plot of medicine formulas. Each column of the icicle plot contains a medicine formula, which comprises principal herbs (text in blue) and other herbs (text in black). The name of the formula is placed under its column.

**Figure 3:**
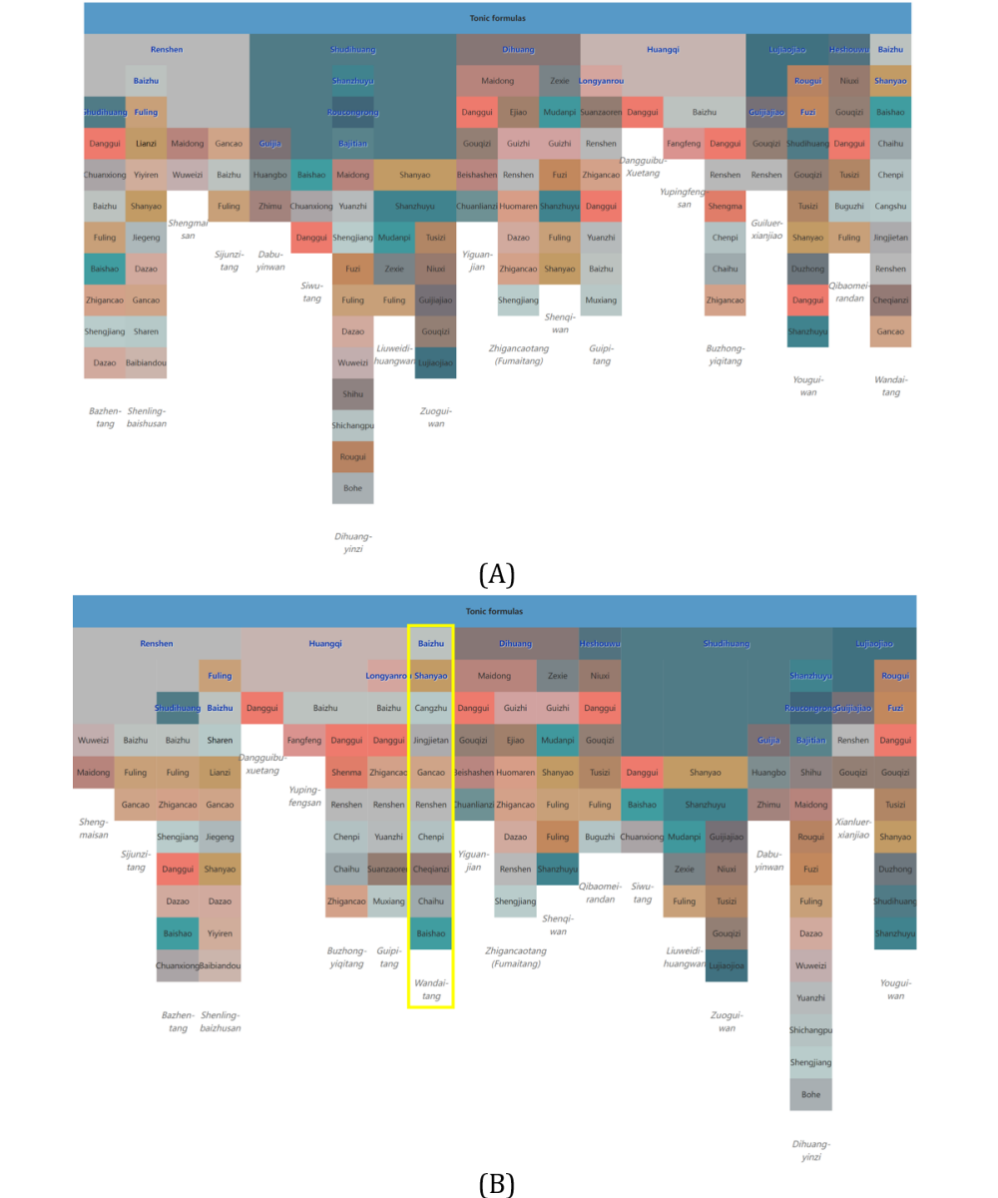
Icicle plots with (A) the original order of medicine formulas data and (B) our similarity-based layout. (This figure is compressed, and a high-resolution version can be found in Multimedia Appendix 2).

Each record in the medicine formula data contains the name of the formula, names of herbs, and tags for principal medicines (Table 1). We set the content of elements of the icicle plot to names of herbs and used each column to show a medicine formula, as shown in Figures 2 and 3.

In our design, principal herbs were highlighted and treated differently from other herbs to meet requirement 3. As shown in Figure 2, principal herbs are placed at the top levels of the hierarchy and colored blue with bold face font and glow. Formulas containing common principal herbs were grouped together. Rows were padded so that the top of all nonprincipal herbs were aligned for comparison (requirement 2). For example, rows are padded for Renshen (Ginseng, 人参), as shown in Figure 2. The name of the medicine formula is placed under its corresponding column in italic font face with a fixed vertical spacing, as shown in Figure 3. This design is simple yet effective: the height of each column is used as an additional cue to the horizontal position for the quick alignment of a formula and its name.

Because the set-based formula information must be converted into columns of the icicle plot, ordering is needed for herbs in a formula. However, herbs in the original data have no specific ordering: the resulting icicle plot of medicine formulas of tonic formulas with the initial ordering of herbs is shown in Figure 3A. The plot is cluttered, and comparing elements of medicine formulas is difficult, as frequent context switch has to be made while searching for the same herb. Therefore, we propose a similarity-based layout method to facilitate an easier comparison and clearer visualization of medicine formulas than using the original ordering.

#### Similarity-Based Layout Computation

#### Overview

Our method is an efficient greedy algorithm with 2 steps based on the similarity of herbs: first, the arrangement of principal herbs and then the arrangement of the remaining herbs.

To facilitate this explanation, we introduced the similarity sequence *S* = (*s*_1_,…,*s_n_*) for a set of herbs *H* = [*h*_1_,…,*h_n_*]. The element *s_i_* in *S* is expressed as follows:



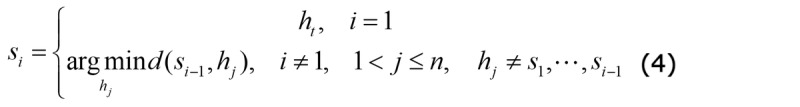



where *d (s, h)* is the distance between *s* and *h* using equation 3 and *t* is a random number between 1 and *n*.

#### Arrangement of Principal Herbs

In this step, the columns of the icicle plot were sorted based on the similarity of the principal herbs. If an herb is the only principal herb in a certain medicine formula, it is assigned as the top-level principal herb. Such herbs of all formulas were sorted using equation 4.

We then treat formulas with ≥1 principal herb. If any principal herb of the formula appears in the top-level principal herb list, it is denoted as the top-level principal herb of that formula; if none of the principal herbs in a formula is contained in the list, a random herb is selected and added to the list. An example is Wandaitang (完带汤) as highlighted in the yellow box in Figure 3. The sorted top-level principal herbs were placed on the first row of the icicle plot. Other principal herbs were sorted according to their distance and laid out as subsequent children nodes as rows with an increasing number of herbs from left to right. To align nonprincipal herbs across formulas for easy comparison, rows of principal herbs were added.

The results after the arrangement of the principal herbs are shown in Figure 2 (a zoomed-in part of Figure 3). Here, Renshen (Ginseng, 人参) is the top-level principal herb, and Shenlingbaizhusan (参苓白术散) and Bazhentang (八珍汤) have ≥1 principal herb (columns 2 and 3, respectively). The principal herb rows are padded to 3, as Shenlingbaizhusan has a maximum of 3 principal herbs.

#### Arrangement of Remaining Herbs

Next, the remaining herbs were arranged. From left to right, each formula column was converted from a set to a sequence. The leftmost column is sorted by distance-based ordering using equation 4. Starting from the second column from the left, medicines are sorted by local similarity—the same herbs in adjacent columns are aligned first, and other herbs are sorted based on distances to the adjacent herbs to the left.

Figure 3B shows the icicle plot of tonic formulas with the new similarity layout. Compared with the original layout (Figure 3A), the alignment of herbs was improved, and the same herbs in adjacent columns were aligned vertically. For example, note how Baizhu (rhizome of Largehead Atractylodes, 白术), Fuling (Indian Bread, 茯苓), and Renshen (Ginseng,人参) are aligned as nonprincipal herbs in Figure 3B, whereas in Figure 3A, such alignments are nonexistent.

### Visualization of Shared Herbs in Formulas

A co-occurrence matrix view of formulas is included to complement the icicle plot for comparing formulas that are far apart, for example, having different principal herbs. The benefit of using a matrix view is that all formulas’ complete pair-wise intersection information can be effectively represented and easily identified.

**Figure 4:**
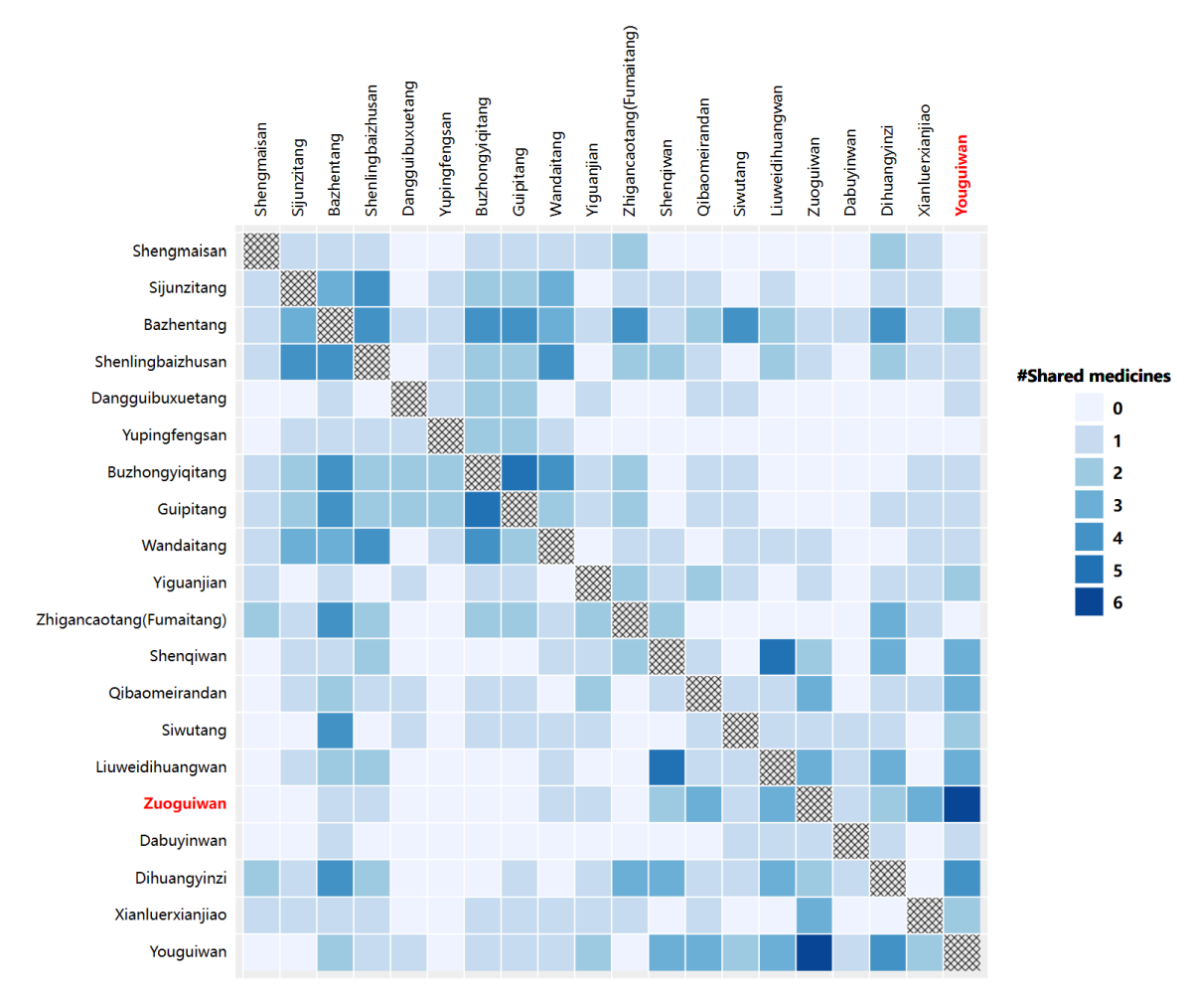
The shared herbs matrix view of formulas.

As shown in Figure 4, the matrix contains formulas as rows and columns and the number of shared herbs as the element value. With a sequential color map, this view allows the user to quickly examine the overlapping information of each formula against all others by focusing on a row or column. In addition, the color encoding effectively draws the attention of the user to formulas with the highest number of shared herbs: in this case, Zuoguiwan (左归丸) and Youguiwan (右归丸) as highlighted in red in Figure 4.

### Perceptual-Guided, Data-Driven Color Encoding

#### Overview

**Figure 5:**
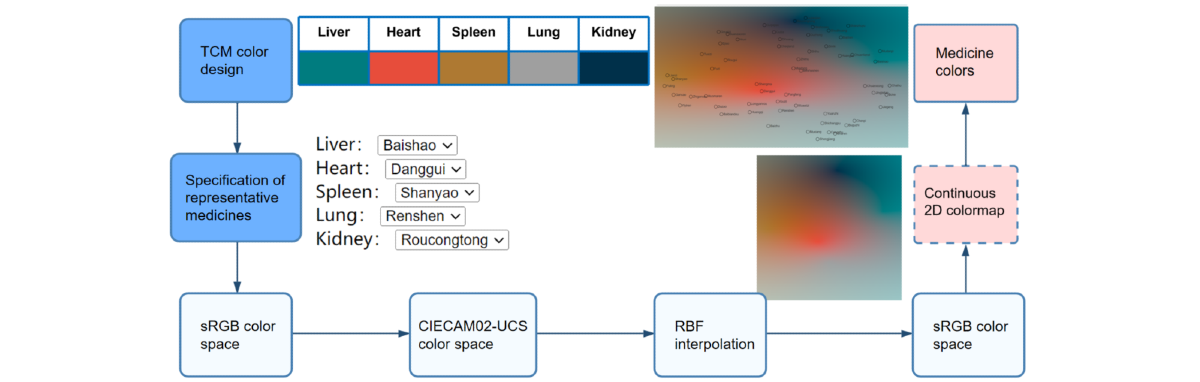
The pipeline of our color-encoding method. CIECAM02-UCS: International Commission on Illumination Color Appearance Model 2002 Uniform Color Space; RBF: radial basis function; sRGB: standard RGB; TCM: traditional Chinese medicine.

The herb and formula views are color encoded based on the multidimensional attributes of herbs with perceptual guidance of their similarity. The workflow of our color-encoding method is illustrated in Figure 5: the method is based on the 2D reduced space derived from multidimensional herb attribute data and requires the knowledge of users to identify representative herbs within it. For a group of herb formulas, medical experts can identify several representative herbs based on their TCM attributes using TCM concept-inspired colors (representative 7). These colors are transformed into a perceptual uniform color space and interpolated with radial basis functions (RBFs) to obtain the herb colors and the continuous 2D color map that spans the entire dimensionality-reduced attribute space.

#### TCM Concept-Inspired Representative Color Design

**Figure 6:**
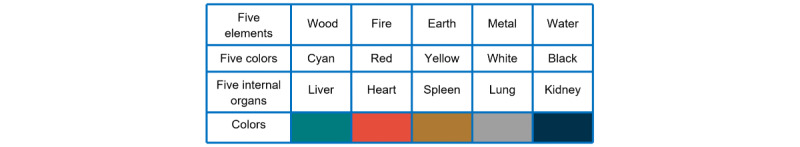
Colors designed for medicine based on traditional Chinese medicine concepts.

The colors of the representative herb were carefully chosen to show TCM concepts. These TCM concepts include 5 elements (五行), 5 colors (五色), and 5 internal organs (五脏), as summarized in Figure 6. The associated colors are handpicked to show the connection to the 5 colors with perceptual and esthetic considerations—the luminance of colors should not vary too much, and saturated colors should be avoided.

#### Perceptual Uniform Color Space

For perceptual uniformity, we used the International Commission on Illumination Color Appearance Model 2002 Uniform Color Space (CIECAM02-UCS) [12] to calculate the colors of the remaining herb with color interpolation. As shown in Figure 5, we transformed the colors of the representative herb from standard RGB (sRGB) to CIECAM02-UCS through the International Commission on Illumination XYZ color space (CIEXYZ). Then, RBF interpolation was performed for each channel of the CIECAM02-UCS. Next, the interpolated colors are converted back to sRGB for display.

#### RBF Color Interpolation

RBF interpolation enables the interpolation of unstructured data, for example, a few scattered points or point clouds, making them a good choice for our method. We experimented with several RBFs, including Gaussian, cubic, and thin-plate functions and chose the linear RBF. The choice is made for 2 reasons: first, the measure of Euclidean distance matches the distance of herbs, and second, the least duplicate colors are generated among the RBFs we tested.

#### Color Assignment

**Figure 7:**
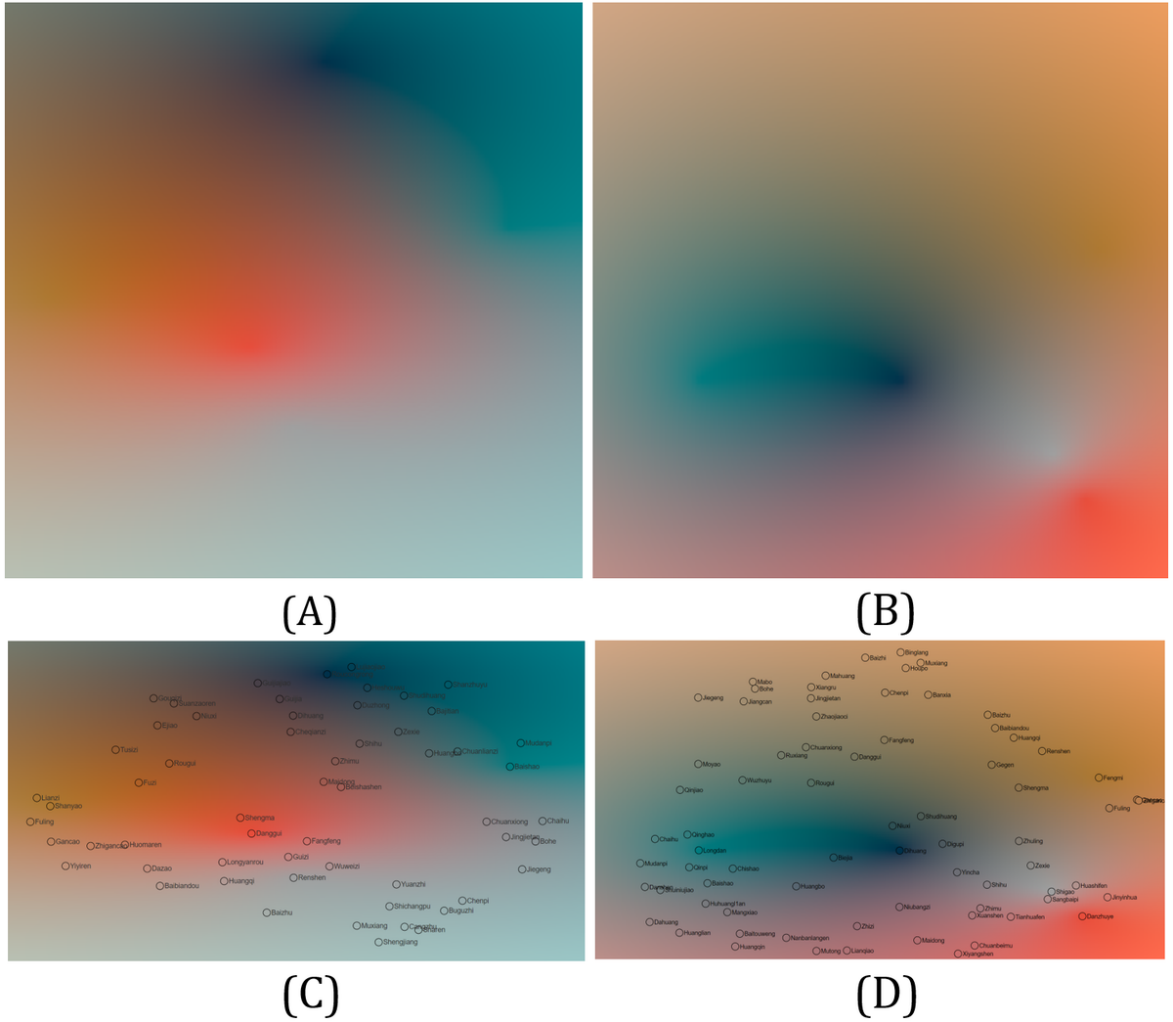
Color encoding with our method for tonic formulas (the left column) and heat-clearing formulas (the right column). Continuous 2D colormaps are shown in parts (A) and (B), respectively. (C) and (D) Herb colors are calculated based on their positions in the 2D domain. (This figure is compressed, and a high-resolution version can be found in Multimedia Appendix 2).

Continuous 2D color maps of the 2 groups of medicine formulas generated by RBF interpolation over the entire 2D domain are shown in Figures 7A and 7B. Smooth transitioning between attributes of medicines can be seen in 2D color maps, whereas color differences indicate distances between medicines. Therefore, 2D continuous color maps are useful tools for examining the distribution of herbs in the multidimensional space of a certain medical formula.

To assign colors to the herbs, the 2D location of each herb in the dimensionality-reduced space was used for the interpolation of colors. Herb colors overlaid on the continuous color map are shown for the 2 formula groups in Figure 7C and 7D. For efficiency, only the colors of points of herbs shown in medicine formulas need to be calculated if the overall trend in the 2D domain is not the focus.

### User Interactions

**Figure 8:**
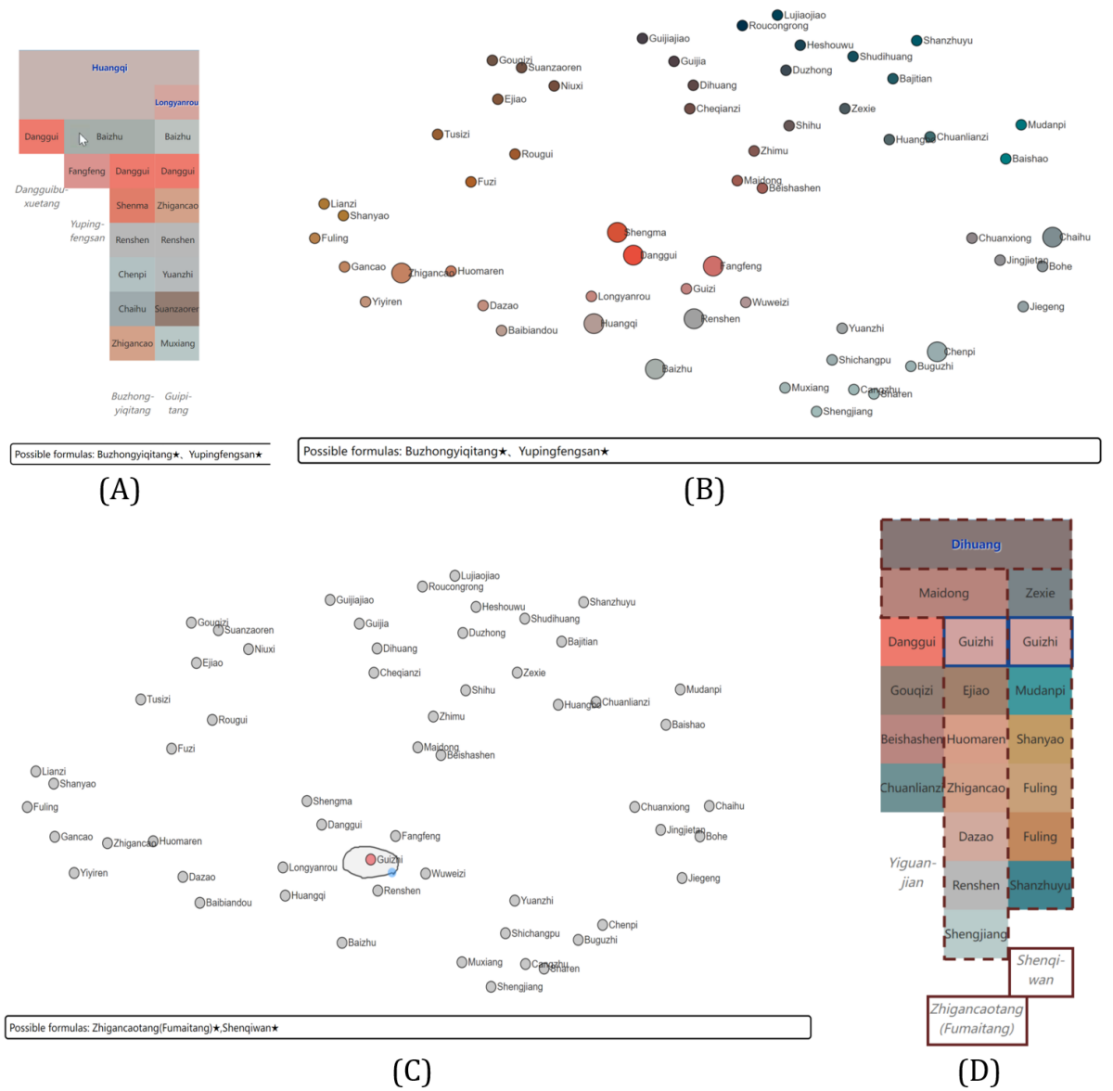
User interactions in our method: (A) mouse hovering in the formula view and (B) corresponding updates in the herb view; (C) lasso selection in the herb view and (D) corresponding changes in the formula view. (This figure is compressed, and a high-resolution version can be found in Multimedia Appendix 2).

**Figure 9:**
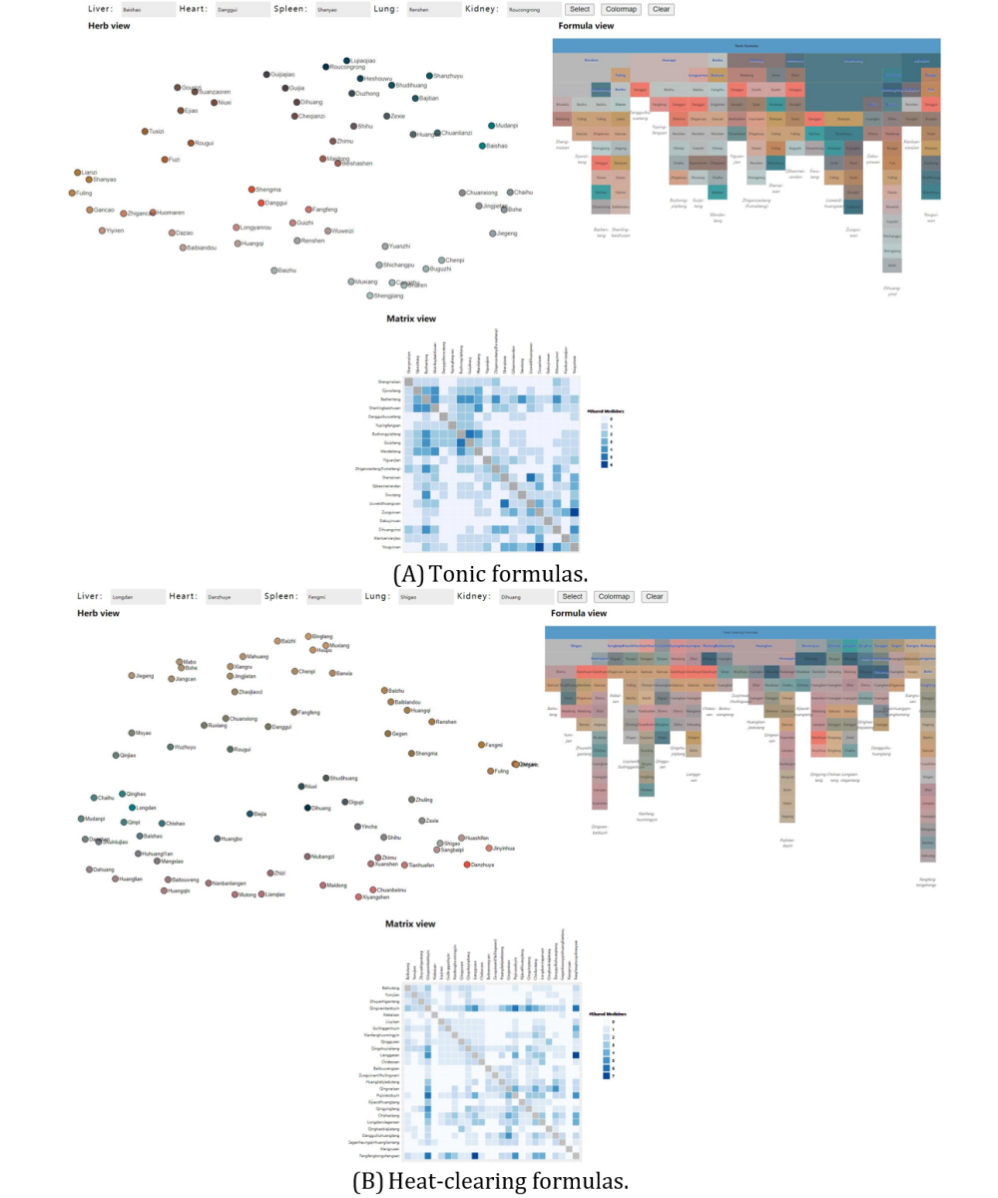
Visualizations of 2 typical groups of medicine formulas with our method: (A) tonic formulas and (B) heat-clearing formulas. (This figure is compressed, and a high-resolution version can be found in Multimedia Appendix 3).

Our visualization method supports interactive exploration within the formula view, the matrix view, and the herb view. Brushing and linking enables connections between these 3 views (requirement 4). In the formula view, the names of all formulas are shown whenever the mouse hovers over an herb, as shown in Figure 8A. The matrix view highlights the corresponding formulas when the mouse hovers over an element. In the herb view, a lasso tool allows users to flexibly select the herbs of interest. All potential formulas are shown as text in the scatterplot of the herb view (Figure 8C). Representative herbs can be assigned and updated through selection boxes on top of the herb view (Figure 9). These user interactions are easy to use and intuitive for users who are not familiar with interactive visualization. Therefore, requirement 6 is satisfied.

Brushing and linking enables visual connections between the formula view and the herb view interactively. All herbs are highlighted in the herb view with enlarged size (Figure 8B) if any formula is selected in the formula view (Figure 8A). Conversely, whenever any herb is selected in the herb view (Figure 8C), the formula view is updated, as shown in Figure 8D. Here, all selected formulas are highlighted with blue solid lines, and formulas containing the selected herbs are highlighted with red dashed lines. As a result, brushing and linking helps enhance the understanding of users regarding the composition of herbs in formulas (requirement 5).

### Implementation

The proposed method was implemented as a web-based visual analysis tool, as shown in Figure 9. Data processing procedures were performed in Python aided by the “umap” package for dimensionality reduction, the “scipy” package for RBF interpolation, and the “color” package for color space transformations. Visualization and user interactions were realized in JavaScript aided by the “D3” package, and the communication between Python and JavaScript components is achieved using the “eel” package.

## Results

### Overview

The evaluation of our method was performed as a usability study with the analysis of 2 representative use cases—tonic and heat-clearing formulas—by 2 TCM experts (SP and XH). They were asked to analyze the formulas using the web-based tool with think-aloud protocol analysis and provide feedback after the session. Both experts were systematically trained in TCM and obtained clinical degrees and certificates in TCM. One has obtained a doctoral degree in TCM (SP), whereas the other has been working in clinical for over 9 years (XH). Both experts have ≥14 years of expertise in TCM.

After introducing our method to the participants, they were asked to explore the medicine formulas data using our visualization tool, whereas the observer observed and talked to the participants. Afterward, they were asked to provide further feedback on the method. Visualizations of the 2 use cases presented to the TCM experts, as in the web-based tool, are shown in Figure 9.

### Statistics of Data Sets for Evaluation

The tonic formulas (Figure 9A) contained 20 formulas and 58 herbs with 17 principal herbs and a median of 1 principal herb per formula. The median number of herbs per formula was 7.5, with a minimum of 2 and maximum of 15. The average number of shared herbs in a pair of formulas was 1.09 (SD 1.22).

The heat-clearing formulas (Figure 9B) contained 26 formulas and 73 herbs with 25 principal herbs with a median of 1 principal herb per formula. The median number of herbs per formula was 6.5, with a minimum of 2 and maximum of 17. The average number of shared herbs between a pair of formulas was 0.98 (SD 1.24).

### Use Cases

Expert PS started the analysis by looking at the overall distribution of herbs and used her knowledge to assign representative herbs for each herb category listed in Figure 6. The resulting continuous 2D colormaps show that the center of the attribute space of tonic formulas is red (Figure 7A), whereas heat-clearing formulas have the center of their space as green and black (Figure 7B). These results indicate the different properties of tonic and heat-clearing formulas and are in line with related TCM concepts.

**Figure 10:**
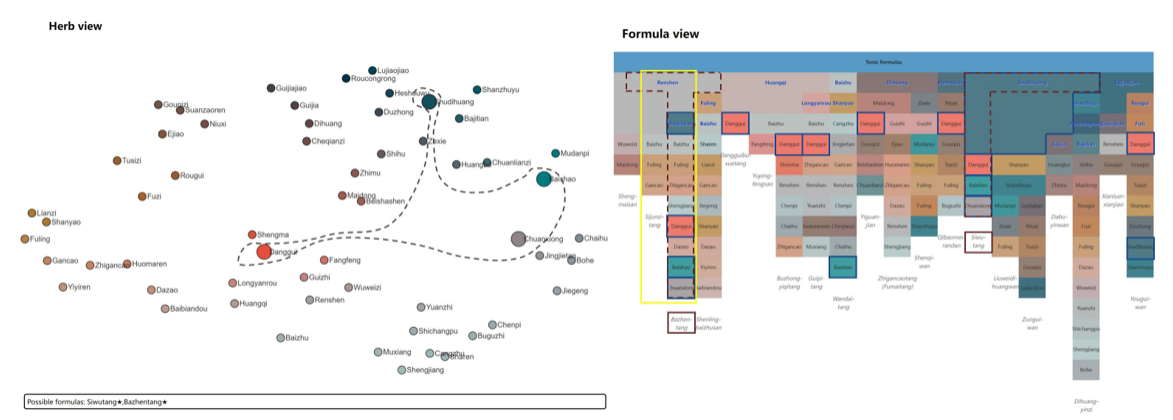
The analysis of tonic prescriptions with our method. A lasso selects 4 herbs of interest in the herb view (left), and corresponding formulas are highlighted in the formula view (right). (This figure is compressed, and a high-resolution version can be found in Multimedia Appendix 4).

In the icicle plot of tonic formulas (Figure 10, right), it is easily seen that 2 adjacent columns are similar: the Bazhentang (八珍汤) contains the Sijunzitang (四君子汤) as highlighted in the yellow box. The TCM expert then analyzed the differences between these 2 formulas. She used the lasso tool in the herb view to select 4 other herbs in Bazhentang, as shown in Figure 10 (left). The text below the scatterplot shows that formulas containing these herbs are Bazhentang and Siwutang (四物汤). These 2 formulas were selected with red dashed lines, and the selected herbs are highlighted with solid blue lines in the formula view (Figure 10, right). A close examination showed that the lasso-selected herbs form Siwutang. Moreover, it can be seen that Bazhentang is the combination of Sijunzitang and Siwutang.

In the matrix view (Figure 9A, right), most formulas have overlapping herbal herbs with Sijunzitang (四君子汤) and Bazhentang (八珍汤), suggesting that tonic formulas are built on the herb composition of these 2 formulas.

It is known that the main role of Sijunzitang or Bazhentang is “invigorating Qi and blood.” The understanding of Qi and blood in TCM is the basic substance of the human body, which can reflect the importance of all supplements to Qi and blood in the matrix view. Yin and Yang are 2 interdependent, opposite, complementary, and exchangeable aspects of nature. Qi is Yang (阳, positive), blood is Yin (阴, negative), and Qi and blood are dependent. TCM physicians usually prescribe for diseases in which Qi and blood deviate from balance. The expert considered that this visualization is suitable for beginners to pay attention to the “Qi and blood” supplement for tonic formulas.

**Figure 11:**
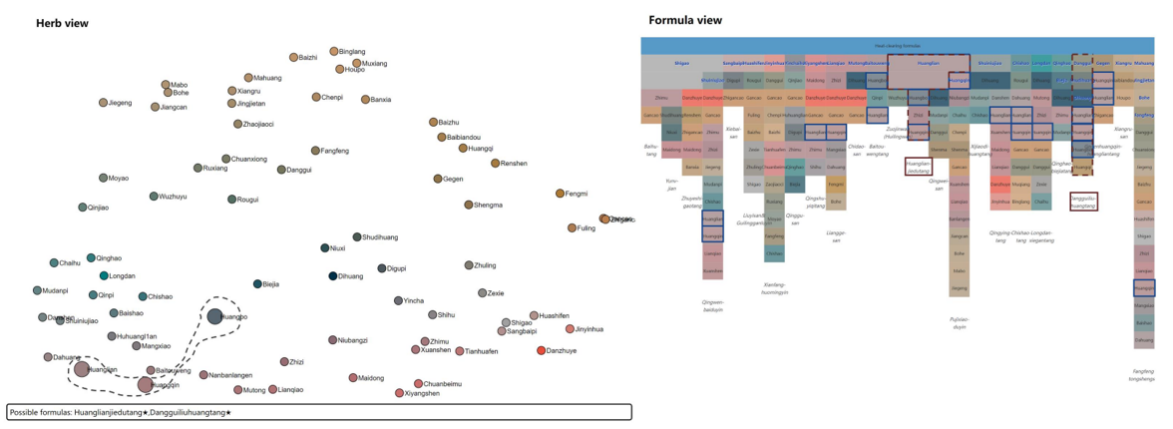
Interactive analysis of heat-clearing formulas with our method. (This figure is compressed, and a high-resolution version can be found in Multimedia Appendix 5).

The analysis of heat-clearing formulas is shown in Figure 11. TCM expert XH was interested in Sanhuang (3 yellow herbs, 三黄): Huanglian (rhizome of Chinese Goldthread, 黄连), Huangqin (root of Membranous Milkvetch, 黄芩), and Huangbo (Phellodendron bark, 黄柏), which is a commonly used herb combination for clearing heat and detoxification in TCM. The 3 herbs were relatively close in the herb view (Figure 11, left), and the expert used a lasso to select them. Both Huanglian-jiedutang (黄连解毒汤) and Danggui-liuhuangtang (当归六黄汤) contain Sanhuang as suggested by the following text. The expert further examined the formula view (Figure 11, right), where these 2 formulas were highlighted. According to the herb attributes, the function of Huanglian-jiedutang is to clear heat and detoxify. Although the composition of Danggui-liuhuangtang contains tonic herbs, meaning that in addition to clearing heat and detoxification, it also has the effect of nourishing Yin (滋阴). Unlike the tonic formulas, not many overlaps are seen in the matrix view (Figure 9B, right). Most formulas have overlapping herbal medicines with Qingwenbaiduyin (清瘟败毒饮), which have the function of clearing heat and detoxification. This can be a reminder for beginners to pay attention to the relationship between this formula and other formulas in the heat-clearing formulas.

### TCM Expert Feedback

Overall, both experts believe that our method can clearly disassemble complex formulas and assist in the memorization of their functionalities. The interactive visual analysis process is new to them and is helpful in enhancing their understanding of formula composition theories by making and testing their own hypotheses. They believe that the color encoding of herbs allows TCM students and beginners to understand the effect of herbs more intuitively and facilitate memorization. Beginners have difficulty understanding the similarities and differences between multiple similar formulas. With the lasso tool, beginners can test multiple herb combinations to better understand the similarities and differences between formulas and, therefore, better understand an actual prescription. In addition, they consider brushing and linking to be a beginner-friendly way to understand the relationships between herbs and formulas. Both experts made positive comments on the coloring of herbs. For example, Danggui (root of Chinese Angelica, 当归) is a blood tonic herb and corresponds to red. On the other hand, Shigao (Gypsum, 石膏) works on the lungs and is colored white.

The experts suggest that in addition to assisting the learning of TCM formulas for beginners, the method can be extended to facilitate the learning of actual treatment plans for TCM physicians. The TCM theory system includes the process of “theory, method, formula, and herb,” and a treatment plan with prescriptions is performed to assess the effectiveness of formulas. The experts suggest supporting multiple lassos as future work to facilitate the building-up of a prescription by adding herbs from an initial known set of herbs to learn actual treatment plans.

## Discussion

### Principal Findings

Our new visualization method could effectively reveal the compositional principle of medicine formulas and assist in the learning of TCM formula composition theories. The proposed method can effectively visualize complex TCM formulas and multidimensional herb attribute information. The joint analysis of medicine formulas and corresponding herbs is possible with user interactions and brushing and linking between multiple views within our web-based tool.

### Comparison With Prior Work

#### Medicine Formulas Analysis and Visualization in TCM

Few specialized visualization methods are available for Chinese medicine formulas analysis. A web-based tool allows for the visualization of formulas, herbal medicines, and photos of herbs [3]. To the best of our knowledge, this approach is the closest to ours: herbal medicines are classified based on their properties within a formula, and the names of herbal medicines are placed in rectangular labels colored by the Jun-Chen-Zuo-Shi attribute. The properties of Siqi, Wuwei, and Guijing are shown as text. However, only 1 formula can be examined at a time, and the visualization is not interactive. Compared with our method, this tool has the advantages of allowing in-depth examination of individual medicine formulas and assisting the recognition of herbs in the real world. Our method is superior to this approach in providing an overview of formulas in a category of prescriptions, allowing interactive exploration and analysis of formulas and herbs and supporting the comparison of herbal medicines with their multidimensional properties.

Cold and hot properties were visualized as indicators of herbal medicine formulas in a formula analysis platform [5]. However, this method covers only 2 properties and does not reveal the multidimensional attributes of herbs. Knowledge graph visualization is proposed for many medicine formulas through manual and natural language processing [4]. In a review paper, a knowledge graph of topics, including medicine formula research, was presented [13]. Network visualization is used to show the composition of medicine formulas to assist in constructing medicine formulas databases [6]. However, these methods do not support the interactive visualization and analysis of formulas, and only partial information of herbal medicine properties is used.

Query-based computer tools without visualization are readily available to assist the learning of herbal medicine formulas. A web-based application allows the searching, browsing, and narration of classic herbal medicine formulas [14]. A tool allows for the recognition of herbs and formulas from prescriptions [15]. Compared with our method, these tools provide complete textual information of herbs and formulas; however, they have neither intuitive visual representation nor the capability to analyze and compare formulas or herbs.

Visualization methods are also used in other research areas of TCM, especially for the diagnosis of phenotypes. For TCM pulse information, visual recognition and visualization have been proposed, and the pulse information is quantified and visualized to support a more accurate diagnosis [16]. Digital tongue images that are important in TCM are recognized and analyzed with a visualization of tongues [17]. Infrared thermal imaging visualization enables users to see and assess physiological states or pathological conditions intuitively, as the temperature of local tissues or the whole body may change owing to illness [18]. Visualization based on a 3D human model of Chinese medicine pulses could facilitate the teaching, understanding, and communication of meridians and acupoints [19]. A visual analysis method for TCM health records has recently become available as a collaboration between TCM and visualization experts [20]. This method supports the analysis of time-varying TCM health records and compares medicines in the formulas of different patients.

#### Visualization Techniques Related to Medicine Formulas Data

Set is an important research subject in visualization. Set visualization techniques were reviewed in a survey by Alsallakh et al [11]. The visualization of set members can be categorized into different strategies, including Euler and Venn diagrams [21-25], node-link diagrams [26-28], matrix-based methods [29-31], and aggregation methods [32,33]. Matrix-based methods support a large number of sets and elements as well as all set relationships. However, the full representation of the matrix is often spatially inefficient for large row or column numbers. In our case, the matrices of sets are sparse; therefore, we used a sparse matrix representation to show the set information, that is, the formulas information, as an icicle plot.

The icicle plot [34] is a popular hierarchical data visualization technique. Hierarchical data visualization techniques can be classified into explicit techniques, that is, trees using node-link diagrams, and implicit techniques that no explicit edges are drawn. Implicit hierarchy visualization techniques were summarized in an extensive survey [35]. The main benefit of implicit techniques is the efficient use of space, making them more suitable for large hierarchical data than trees. Popular implicit methods include treemaps [36,37] and icicle plots [34]. With our augmented icicle plot with a similarity-based layout, our TCM experts consider it easy to understand and allow for quick comparison of formulas.

Multidimensional data can be effectively visualized using dimensionality-reduction techniques [38]. Nonlinear dimensionality-reduction methods [39] are more suitable for preserving complex high-dimensional structures than linear methods [40]. Currently, T-distributed Stochastic Neighbor Embedding (t-SNE) [41] and UMAP [10] are the most popular nonlinear dimensionality-reduction methods because they could preserve the neighboring information in the high-dimensional space. We chose UMAP in our method because it is more efficient and overcomes several limitations of t-SNE.

#### Perceptual Color Spaces

Color perception is important for visualization. A survey of the use of colors in visualization can be found elsewhere [42]. A key concept for the effective use of colors is perceptual uniformity, that is, the perceived color difference should match the data value difference. Perceptual uniformity is used in color map design [43,44]. To achieve perceptual uniformity, colors have to be computed in a uniform color space. International Commission on Illumination Lab color space (CIELab) is perhaps the most well-known perceptual uniform color space [45]. However, studies have shown that the uniformity performance of CIELab is not satisfactory [12]. Recently, several color spaces based on the International Commission on Illumination Color Appearance Model 2002 [46] with better uniformity than CIELab are available. In our method, we chose the CIECAM02-UCS for its good performance, and we proposed a color-encoding method for drugs based on a 2D color map created by RBF interpolation of colors in the CIECAM02-UCS. Prior techniques, for example, the ColorBrewer tool, which is available for perceptual uniform color map design [47], do not support 2D uniform color maps.

### Limitations

Our method does not directly support the visualization of overlaps of ≥2 medicine formulas, that is, intersections of ≥2 sets. However, such information can be implicitly gained by visual searching in the medicine formula view and by interactively selecting herbs of interest that would highlight all formulas containing shared herbs.

Another limitation is that the dimensional reduction view does not explicitly show multidimensional properties but rather the relative distances between herbs. This could be addressed using additional multidimensional visualization techniques, such as parallel coordinates.

### Future Work

In the future, we would like to further enhance the comparison capability of our method. For example, we could support comparing multiple formulas that are not adjacent and apply set visualization techniques to show the correspondence of medicines and formulas directly in the herb view.

Moreover, we would like to apply their method to analyze more groups of formulas and TCM prescriptions in a clinical setting to assist TCM students and doctors to enhance their understanding of formula composition theories and improve their practice.

### Conclusions

We introduced a visualization method for TCM formulas. The requirements and design choices of our method are made through a close collaboration between visualization and TCM experts in an iterative, quick-prototyping fashion. Our method supports interactive visualization of medicine formulas with a similarity-based layout complemented by a matrix view of shared herbs by formulas, and multidimensional attribute data of herbs are visualized using a dimensionality-reduction method. The colors of visual elements are assigned with a perceptual-guided, data-driven color-encoding method that achieves perceptual uniformity and reflects TCM concepts. The web-based tool that implements our method supports the interactive analysis and comparison of medicine formulas and corresponding herbs with brushing and linking between different views. The usability study of our method with TCM experts demonstrated the effectiveness of our method for joint TCM formula composition and herb property analysis. Further feedback from experts suggests that our method has potential for educating TCM formula composition theories and modernizing TCM inheritance methods.

## Appendix

Multimedia Appendix 1 The supplementary material of technical details and herb names conversion table for Chinese, Pinyin, English, and Latin.

Multimedia Appendix 2 High resolution figures.

Multimedia Appendix 3 High-resolution version of fig 9.

Multimedia Appendix 4 High-resolution of fig 10.

Multimedia Appendix 5 High-resolution version of fig 11.

## Abbreviations

CIECAM02-UCS: International Commission on Illumination Color Appearance Model 2002 Uniform Color Space
CIELab: International Commission on Illumination Lab color space
RBF: radial basis function
sRGB: standard RGB
TCM: traditional Chinese medicine
t-SNE: T-distributed Stochastic Neighbor Embedding
UMAP: uniform manifold approximation and projection for dimension reduction
