# Supplementary material for: Visualization of Traditional Chinese Medicine Formulas: Development and Usability Study
Source: JMIR Form Res. 2023 Apr 21;7:e40805. doi: 10.2196/40805 (PMC10163399; doi:10.2196/40805)
Supplement: Multimedia Appendix 1 [file formative_v7i1e40805_app1.docx]

# **Visualization of Traditional Chinese Medicine Formulas—Supplemental Material**

### This supplemental material contains contents left out from the paper for conciseness. Specifically, we provide technical details of each module of the visualization method and a conversion table of herb names between Chinese characters, Pinyin, English, and Latin.

### Dimensionality Reduction and Distance Computation

The attributes of an herbal medicine can be written as an M-dimensional (M=23) vector **P** of binary valued elements:

$$\mathbf{P}=[a_{1},a_{2},\ldots,a_{\text{M}}]^{T}, a_{i}\in\{0,1\} ,i\in[1,\text{M}] .$$

The M-dimensional space is then dimensionality reduced to 2D with a vector **p** of real values:

$\mathbf{p}=[a_{1}^{*},a_{2}^{*}]^{T}, a_{1}^{*},a_{2}^{*}\in[0,1]$.

UMAP[1] is used for its structure preservation ability and computational efficiency.

The distance between herbs is the basis of our subsequent similarity-based layout computation and visualization. We define the distance *d(u, v)* between two herbs *u* and *v* as the L2-norm, i.e., Euclidean distance, between their corresponding 2D vectors **p_u_** and **p_v_**, respectively:

$d\left(u,\text{ }v\right)=||\text{p}_{\text{u}}-\text{p}_{\text{v}}||$.

The distance between **P_u_** and **P_v_** in the original M-dimensional space is also considered. However, our experiment shows that the difficulty of discriminating herbs based on the distance with **P** is higher than with the projected vectors **p**, and the resulting visualization based on **P** is more difficult for comparison and comes with more visual clutter.

### Formulas Visualization

#### Domain Expert Evaluation of Set Visualization Methods

We performed an evaluation of popular sets visualization techniques for the design of a proper set visualization method with the TCM expert (SP). Figures of a Euler diagram, a node-link diagram, and matrix-based methods included in a set visualization survey paper [2] were shown to the TCM expert. The expert was asked to rank the feasibility of these methods for medicine formulas visualization based on the scalability, the ease of understanding, and the support of comparison. The matrix-based method is ranked first by the TCM expert, followed by the node-link diagram, the Euler diagram, and the overlay herbs.

Based on this informal evaluation, we decided to devise a sparse matrix-based method based on the evaluation to show formulas and corresponding medicines to meet requirements R1 and R2. To support the analysis of overlapping herbs within formulas, a co-occurrence matrix view is used to complement the formulas view.

#### Icicle Plot of Medicine Formulas

Our formula-medicine matrix (set-element matrix) treats formulas (sets) as columns and herbs (elements) as rows. The matrix can be shown with a sparse representation as a collection of formula columns of their corresponding herb rows. This representation is similar to an icicle plot for hierarchy visualization. It has the potential to support the comparison of similar medicine formulas If properly laid out. Furthermore, the icicle plot allows for encoding herbs in a hierarchy to separate principal herbs and other herbs.

Each record in the medicine formula data contains the name of the formula, names of herbs, and tags for principal medicines (Table [1).](#_bookmark1) We set the content of elements of the icicle plot to names of herbs, and use each column to show a medicine formula as shown in Figures [2](#_bookmark9) and [3.](#_bookmark10)


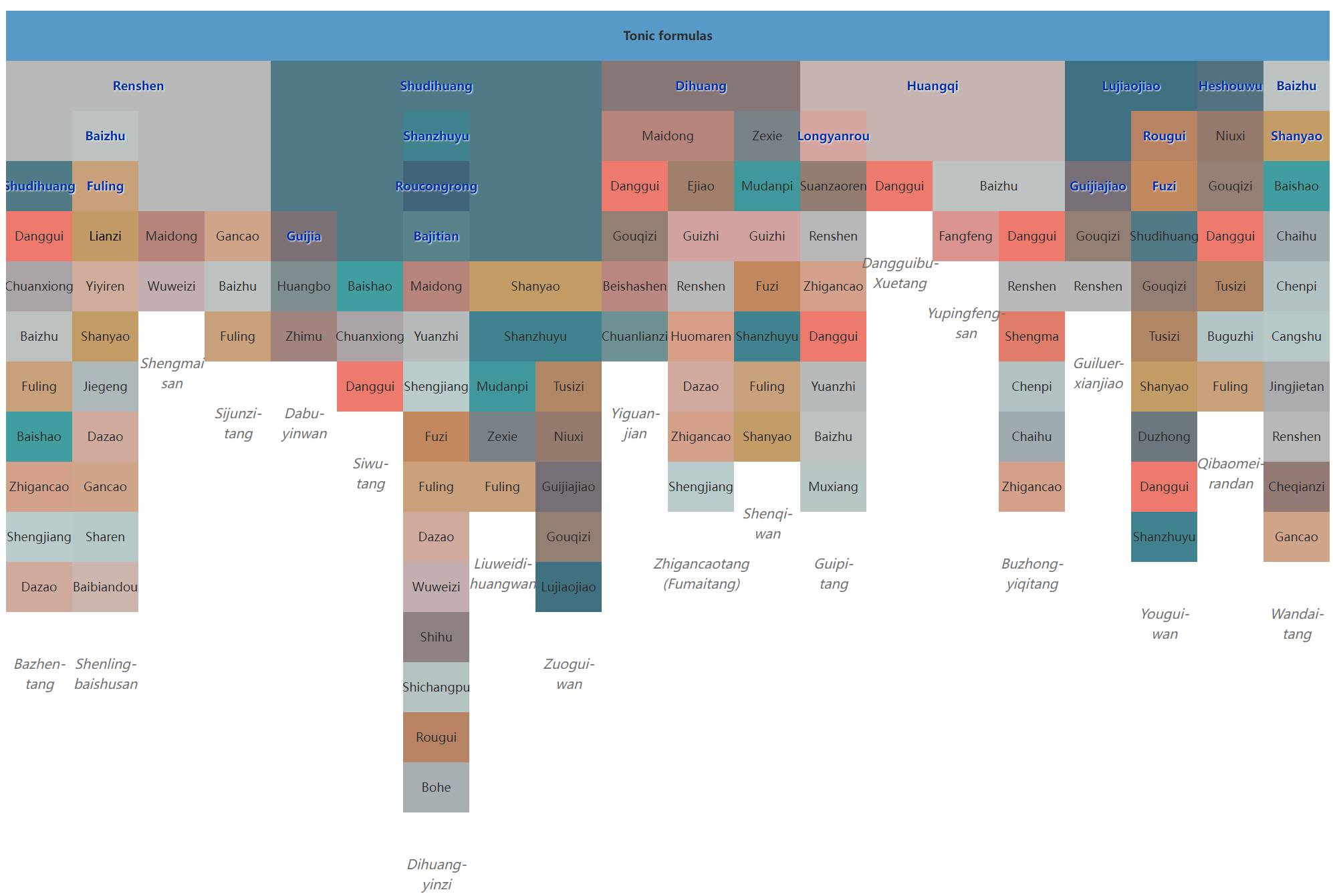

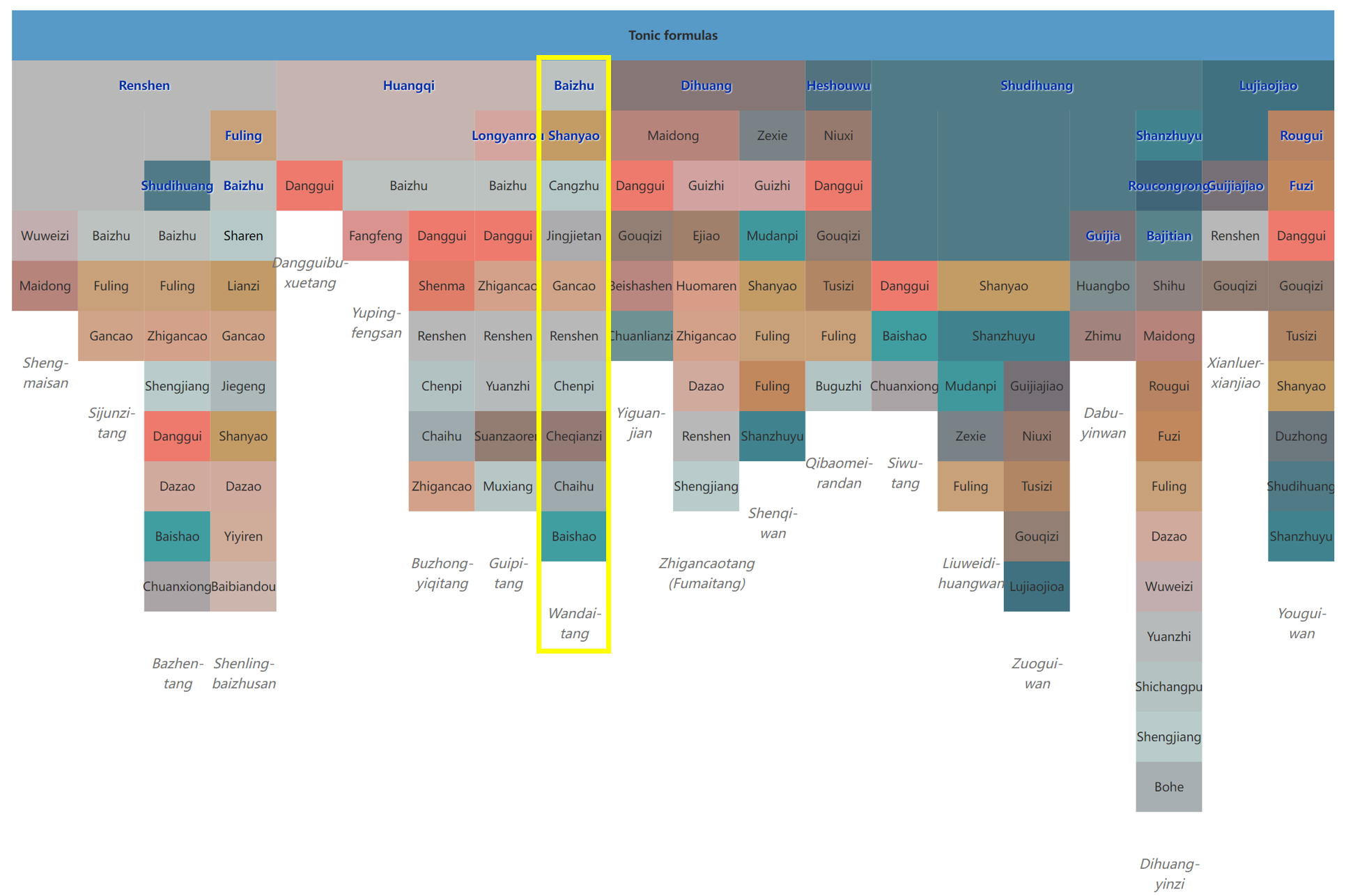


1. (b)

Fig. 2: Icicle plots with (a) the original order of medicine formulas data and (b) our similarity-based layout.


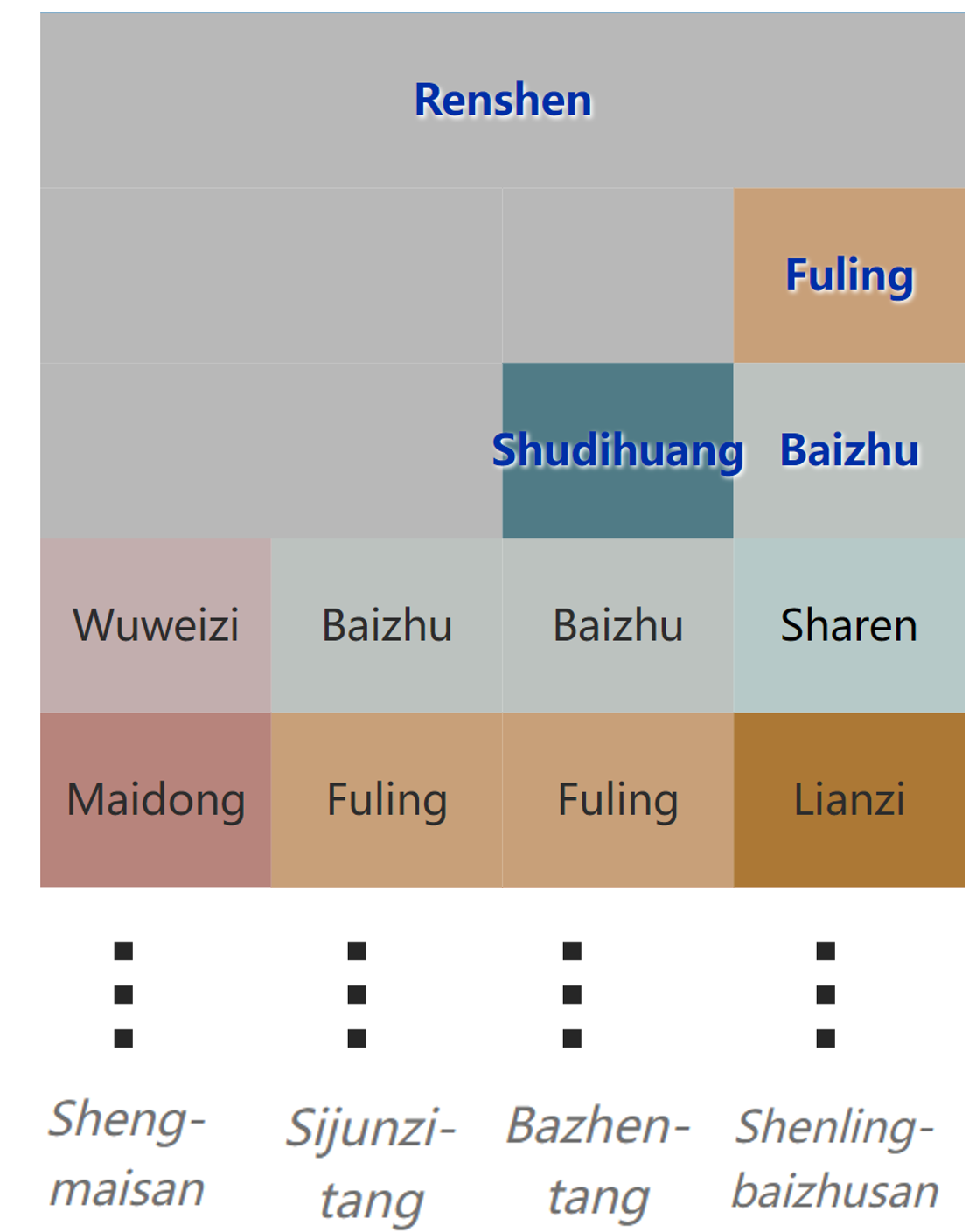


Fig. 3: The design of the icicle plot of medicine formulas. Each column of the icicle plot contains a medicine formula, which comprises principal herbs (texts in blue) and other herbs (texts in black). The name of the formula is placed under its column.

In our design, principal herbs are highlighted and treated differently than other herbs to meet requirement R3. As shown in Figure 3, principal herbs are placed on the top levels of the hierarchy and colored blue with bold face font and glow. Formulas with common principal herbs are grouped together. Rows are padded so that the top of all none-principal herbs are aligned for comparison (R2). For example, rows are padded for Renshen (人参) as seen in Figure 3. The name of a medicine formula is placed under its corresponding column in italic font face with a fixed vertical spacing as shown in Figure 2. This design is simple yet effective: the height of each column is used as an additional cue to the horizontal position for quick alignment of a formula and its name.

Since the set-based formula information has to be converted into columns of the icicle plot, an ordering is needed for herbs in a formula. However, herbs in the original data have no specific ordering: the resulting icicle plot of medicine formulas of tonic formulas with the initial ordering of herbs has been shown in Figure 2 (a). The plot is cluttered and comparing elements of medicine formulas is difficult as frequent context switch has to be made while searching for a same herb. Therefore, we propose a similarity-based layout method to facilitate easier comparison and clearer visualization of medicine formulas than using the original ordering.

#### Similarity-Based Layout Computation

Our method is an efficient greedy algorithm with two steps based on the similarity of herbs: first, the arrangement of principal herbs, and then we arrange the remaining herbs.

To facilitate the explanation, we introduce the similarity sequence $S=\left(s_{1},\cdots,s_{n}\right)$ for a set of herbs $H=\left\{h_{1},\cdots,h_{n}\right\}$. The element *s_i_* of *S* reads:

$$s_{i}=\left\{\begin{matrix} h_{t}, i=1 \\ \mathop{\mathrm{argmin}}\limits_{h_{j}} d(s_{i-1},h_{j}), i\neq1, 1<j\leq n, h_{j}\neq s_{1},\cdots,s_{i-1} \end{matrix}\right.$$

where $d(s,h)$ is the distance between *s* and *h* with Equation 3, and *t* is a random number between 1 and *n*.

**Arrangement of Principal Herbs**

In this step, columns of the icicle plot are sorted based on the similarity of principal herbs. If an herb is the only principal herb in a certain medicine formula, it is assigned as the top-level principal herb. We denote the set of all such herbs as *H_s_*. The first element *s_1_ = h_t_* is randomly selected from *H_s_*, and the rest of the sequence is set by finding the herb *h_j_* with the shortest distance to the previously ordered element *s_i_*. The sorted top-level principal herbs are placed on the first row of the icicle plot.

We now process formulas with more than one principal herb. For a top-level principal herb *h_i_*, a set $\Omega_{i}$ denotes all medicine formulas that have *h_i_* as a principal herb, and the *j*-th formula in $\Omega_{i}$ is denoted as $\Omega_{ij}$. If its principal herb contains elements of *H_s_*, add that formula to set $\Omega_{i}$; if none of the principal herbs in a formula is contained in *H_s_*, *h_j_* is selected randomly as the top-level herb and added to *H_s_*. An example is Wandaitang (完带汤) in Figure 2.

For each $\Omega$, formulas with single principal herb are sorted from left to right by the number of remaining herbs; the number of principal herbs sorts formulas with multiple principal herbs. Principal herbs that are not top-leveled are sorted according to the distance and laid out as subsequent children nodes (as rows). The padding ensures that all non-principal herbs start on the same row.

For example, Figure 3 visualizes set $\Omega_{1}$ of Figure 2, with Ginseng (人参) as the top-level principal herb, and Bazhentang (八珍汤) and Shenlingbaizhusan (参苓白术散) have more than one principal herbs (columns 2 and 3, respectively). Therefore, the principal herb rows are padded to three rows as Shenlingbaizhusan has a maximum of three principal herbs.

**Arrangement of Remaining Herbs**

Next, the remaining herbs are arranged. Define the herb in the *j*-th column, and *k*-th row in the *i*-th multiple medicine formulas set $\Omega_{i}$ in the icicle plot as *h_ijk_*. Each formula column is then the previously introduced set$\Omega_{ij}=\left\{h_{ij1},\cdots,h_{ijm}\right\}$. We construct the position sequence of $\Omega_{ij}$ as $P_{ij}=(p_{ij1},\cdots,p_{ijm})$ from left to right. The leftmost column is sorted by the distance-based ordering using Equation 3. Starting from the second column from the left, medicines are sorted by local similarity. We align the same herbs in adjacent columns even if they are not from the same $\Omega$. Other herbs are sorted according to their distances to herbs on its left column within the set $\Omega_{i}$:

$$p_{ijk}=\left\{\begin{matrix} p_{i(j-i)k}, p_{i(j-1)k}\subset\Omega_{ij} \\ \mathop{\mathrm{argmin}}\limits_{h_{ijl}} d(p_{i(j-1)k},h_{ijl}), 1<l\leq m, h_{ijl}\neq p_{ij1},\cdots,p_{ijk-1}\text{，}h_{ijl}\neq p_{i(j-i)k}\text{（}p_{i(j-1)k}\subset\Omega_{ij}\text{）} \end{matrix}\right.$$

If $\Omega_{ij}$ contains more elements than $\Omega_{i\left(j-1\right)}$, construct the hierarchy with similarity ordering as in Equation 4.

Figure 2 (b) shows the icicle plot of tonic formulas with the new similarity layout. Compared to the original layout (Figure 2 (a)), the alignment of herbs is improved and the same herbs in adjacent columns are aligned vertically. For example, note how Baizhu (白术), Fuling (茯苓), and Ginseng (人参) are aligned as non-principal herbs in Figure 3 (b), whereas in Figure 2 (a), such alignments are nonexistent.

### Perceptual-Guided Data-Driven Color Encoding

#### TCM-Concept Inspired Representative Color Design

Colors of representative herb are carefully chosen to show TCM concepts. These TCM concepts include Five elements (五行), Five colors (五色), and Five internal organs (五脏) as summarized in Figure 6. The associated colors are handpicked to show the connection to Five colors with perceptual and aesthetic considerations—the luminance of colors should not vary too much, and saturated colors should be avoided. Initially, we experimented with isoluminant colors that are beneficial for metric comprehension. However, the TCM expert considers the resulting colors are not distinct enough in the herb view. As a trade-off, the representative colors are chosen to have a relatively small range of luminance (14 ≤Y ≤ 62).


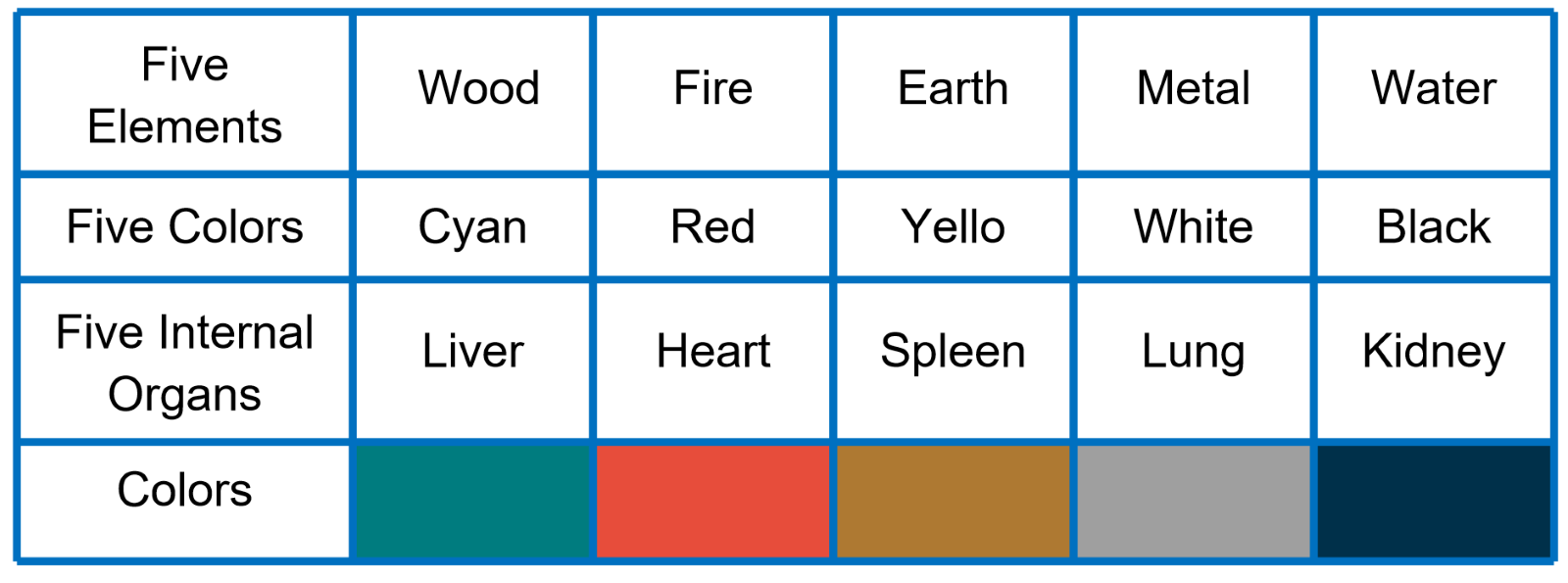


Fig. 6: Colors designed for medicine based on TCM concepts.

#### Perceptual-Uniform Color Space

For perceptual uniformity, we use the CIECAM02-UCS color space [3] to calculate colors of the remaining herb with color interpolation. This color space is spanned by three parameters $(J,a*,b*)$ that is calculated by first transforming XYZ color stimuli to the $(J,c,h)$channels of the CIECAM02 color appearance model followed by a transformation from CIECAM02 to CIECAM02-UCS. As shown in Figure 5, we transform the colors of representative herb from sRGB to CIECAM02-UCS through CIEXYZ. Then, the RBF interpolation is performed for *J*,$a*$and $b*$ channels, respectively. Next, the interpolated colors are converted back to sRGB for display.

#### Radial Basis Function Color Interpolation

Radial basis function (RBF) interpolation enables the interpolation of unstructured data, e.g., a few scattered points or point clouds—making them a natural choice for our method. The RBF interpolation function s(**x**) at location **x** can be written as:

,

where *K* is the number of known data points (*K* = 5 according to Figure 6), **x***_k_* are the locations of known data points, is the radial basis function of a distance r between **x** and **x***_k_* (here, the Euclidean distance), and *w_k_* are unknown weights. If **x** = **x***_k_* ,$k\in[1,2,\ldots,K]$, interpolated values should be data values y at that location: s(**x**) = f (**x**) = y.

We experimented with several radial basis functions, including, Gaussian, cubic, thin-plate functions, and choose the linear radial basis function:

.

The choice is made due to two reasons: first, the measure of Euclidean distance which matches the distance of herbs, and second, it also generates least duplicate colors among typical radial basis functions.

### References

1. McInnes L, Healy J, Melville J. UMAP: Uniform Manifold Approximation and Projection for Dimension Reduction. Published online 2018.

2. Alsallakh B, Micallef L, Aigner W, Hauser H, Miksch S, Rodgers P. The State-of-the-Art of Set Visualization. *Computer Graphics Forum*. 2016;35(1):234-260. doi:10.1111/cgf.12722

3. Luo MR, Cui G, Li C. Uniform colour spaces based on CIECAM02 colour appearance model. *Color Research \& Application*. 2006;31(4):320-330. doi:https://doi.org/10.1002/col.20227

**Table of Herb Names**

| **Chinese_name** | **Pinyin_name** | **Latin_name** | **English_name** |
| --- | --- | --- | --- |
| **阿胶** | Ejiao | Asini Corii Colla | Ass-hide Gelatin |
| **巴戟天** | Bajitian | Morindae Officinalis Radix | Morindae Officilis Radix |
| **白扁豆** | Baibiandou | Semen Lablab Album,Lablab Semen Album | White Hyacinth Bean |
| **白芍** | Baishao | Radix Paeoniae Alba,Paeoniae Radix Alba | White peony root |
| **白术** | Baizhu | Atractylodis Macrocephalae Rhizoma,Rhizoma Atractylodis Macrocephalae | rhizome of Largehead Atractylodes |
| **白头翁** | Baitouweng | Pulsatillae Radix | Root of Chinese Pulsatilla |
| **白芷** | Baizhi | Angelicae Dahuricae Radix | Dahurian Angelica Root |
| **板蓝根** | Banlangen | Isatidis Radix | Root of Dyers Woad |
| **半夏** | Banxia | Pinelliae Rhizoma | tuber of Pinellia |
| **薄荷** | Bohe | Menthae Haplocalycis Herba,Herba Menthae | Mentha, Peppermint |
| **北沙参** | Beishashen | Radix Glehniae,Glehniae Radix | root of Coastal Glehnia |
| **鳖甲** | Biejia | Trionycis Carapax | Turtle Carapace |
| **槟榔** | Binglang | Arecae Semen | areca seed |
| **补骨脂** | Buguzhi | Psoraleae Fructus | Malaytea Scurfpea Fruit |
| **苍术** | Cangzhu | Atractylodis Rhizoma,Rhizoma Atractylodis | Rhizome of Swordlike Atractylodes |
| **柴胡** | Chaihu | Radix Bupleuri,Bupleuri Radix | root of Chinese Thorowax |
| **车前子** | Cheqianzi | Semen Plantaginis,Plantaginis Semen | seed of Asiatic pantain |
| **陈皮** | Chenpi | Citri Reticulatae Pericarpium,Pericarpium Citri Reticulatae | Dried Tangerine Peel |
| **赤芍** | Chishao | Paeoniae Radix Rubra | Red Peony root |
| **川贝母** | Chuanbeimu | Fritillariae Cirrhosae Bulbus | Bulb of Tendrilleaf Fritillary |
| **川楝子** | Chuanlianzi | Toosendan Fructus,Fructus Meliae Toosendan | Szechwan Chiberry Fruit |
| **川芎** | Chuanxiong | Chuanxiong Rhizoma | Chuanxiong rhizome, Szechuan lovage root |
| **大黄** | Dahuang | Rhei Radix Et Rhizoma | root and rhziome of Sorrel Rhubarb |
| **大枣** | Dazao | Fructus Zizyphi Jujubae,Jujubae Fructus | Jujube Chinese date |
| **丹参** | Dahuang | Rhei Radix Et Rhizoma | root and rhziome of Sorrel Rhubarb |
| **淡竹叶** | Dahuang | Rhei Radix Et Rhizoma | root and rhziome of Sorrel Rhubarb |
| **当归** | Danggui | Radix Angelicae Sinensis,Angelicae Sinensis Radix | root of Chinese Angelica |
| **地骨皮** | Digupi | Lycii Cortex | root - bark of Chinese Wolfberry |
| **地黄** | Dihuang | Rehmanniae Radix | Rehmannia Glutinosa |
| **杜仲** | Duzhong | Cortex Eucommiae,Eucommiae Cortex | Eucommia bark |
| **防风** | Fangfeng | Radix Saposhnikoviae Divaricatae,Saposhnikoviae Radix | root of Divaricate Saposhnikovia |
| **蜂蜜** | Fengmi | Mel | Honey |
| **茯苓** | Fuling | Poria | Indian Bread |
| **附子** | Fuzi | Aconiti Lateralis Radix Praeparata,Radix Aconiti Lateralis | Common Monkshood Daughter Root |
| **甘草** | Gancao | Glycyrrhizae Radix Et Rhizoma,Radix Glycyrrhizae | Root of Ural Licorice |
| **葛根** | Gegen | Puerariae Lobatae Radix | root of lobed kudzuvine |
| **枸杞子** | Gouqizi | Lycii Fructus,Fructus Lycii | fruit of Chinese Wolfberry |
| **龟甲** | Guijia | Testudinis Carapax Et Plastrum | Tortose's Carapae and Plastron |
| **龟甲胶** | Guijiajiao | Testudinis Carapacis Et Plastri Colla | Glue of tortoise shell |
| **桂枝** | Guizi | Cinnamomi Ramulus,Ramulus Cinmomi | Cassia Twig |
| **何首乌** | Heshouwu | Polygoni Multiflori Radix | Tuber Fleeceflower Root |
| **厚朴** | Houpo | Magnoliae Officinalis Cortex | bark of Officil magnolia |
| **胡黄连** | Huhuanglian | Picrorhizae Rhizoma | Picrorhizae Rhizoma |
| **滑石粉** | Huashifen | Talci Pulvis | talc powder |
| **黄柏** | Huangbo | Phellodendri Chinensis Cortex,Cortex Phellodendri | Phellodendron bark |
| **黄连** | Huanglian | Coptidis Rhizoma | rhizome of Chinese Goldthread |
| **黄芪** | Huangqi | Astragali Radix,Radix Astragali | root of Membranous Milkvetch |
| **黄芩** | Huanglian | Coptidis Rhizoma | rhizome of Chinese Goldthread |
| **火麻仁** | Huomaren | Cannabis Fructus,Semen Canbis;Fructus Canbis | Hemp Seed |
| **僵蚕** | Jiangcan | Bombyx Batryticatus | Stiff Silkworm |
| **金银花** | Jinyinhua | Lonicerae Japonicae Flos | Honeysuckle Flower |
| **荆芥炭** | Jingjietan | Schizonepetae Herba Carbonisata | Fineleaf Schizonepeta Herb |
| **桔梗** | Jiegeng | Radix Platycodi,Platycodonis Radix | Platycodon Root |
| **连翘** | Lianqiao | Forsythiae Fructus | Weeping Forsythia Capsule |
| **莲子** | Lianzi | Nelumbinis Semen | Lotus Seed |
| **龙胆** | Longdan | Gentianae Radix Et Rhizoma | Gentiae Radix Et Rhozima |
| **鹿角胶** | Lujiaojiao | Cervi Cornus Colla | Antler gum |
| **麻黄** | Mahuang | Ephedrae Herba | Ephedra |
| **马勃** | Mabo | Lasiosphaera Calvatia | Puff-ball |
| **麦冬** | Maidong | Ophiopogonis Radix | Dwarf lilyturf tuber |
| **芒硝** | Mangxiao | Natrii Sulfas | Natrii Sulfas |
| **没药** | Moyao | Myrrha | Myrrh |
| **牡丹皮** | Mudanpi | Cortex Moutan,Moutan Cortex | Tree Peony Bark |
| **木通** | Mutong | Clematidis Armandii Caulis | Armand Clematis Stem |
| **木香** | Muxiang | Aucklandiae Radix | Common Aucklandia Root |
| **牛蒡子** | Niubangzi | Arctil Fructus | achene of Great Burdock |
| **牛膝** | Niuxi | Radix Achyranthis Bidentatae, Achyranthis Bidentatae Radix | root of Twotooth Achyranthes |
| **秦艽** | Qinjiao | Gentianae Macrophyllae Radix | root of Largeleaf Gentian |
| **秦皮** | Qinpi | Fraxini Cortex | Ash Bark |
| **青蒿** | qinghao | Artemisiae Annuae Herba | Sweet Wormwood Herb |
| **人参** | Renshen | Ginseng Radix Et Rhizoma, Radix Ginseng | Ginseng |
| **肉苁蓉** | Roucongrong | Herba Cistanches, Cistanches Herba | Desertliving Cistanche |
| **肉桂** | Rougui | Cortex Cinmomi, Cinnamomi Cortex | Cassia Bark |
| **乳香** | Ruxiang | Olibanum | Frankincense |
| **桑白皮** | Sangbaipi | Mori Cortex | Cortex Mori |
| **砂仁** | Sharen | Amomi Fructus,Fructus Amomi | Villous Amomum Fruit |
| **山药** | Shanyao | Rhizoma Dioscoreae, Dioscoreae Rhizoma | Common Yam Rhizome |
| **山茱萸** | Shanzhuyu | Fructus Corni,Corni Fructus | Asiatic Cornelian Cherry Fruit |
| **升麻** | Shengma | Cimicifugae Rhizoma, Rhizoma Cimicifugae | Largetrifoliolious Bugbane Rhizome |
| **生姜** | Shengjiang | Zingiberis Rhizoma Recens, Rhizoma Zingiberis Recens | Fresh Ginger |
| **石菖蒲** | Shichangpu | Rhizoma Acori Talarinowii, Acori Tatarinowii Rhizoma | Grassleaf Sweetflag Rhizome |
| **石膏** | Shigao | Gypsum Fibrosum | Gypsum |
| **石斛** | Shihu | Dendrobii Caulis | Noble Dendrobium Stem Herb |
| **熟地黄** | Shudihuang | Rehmanniae Radix Praeparata, Radix Rehmanniae Preparata | Prepared Rehmannia Root |
| **水牛角** | Shuiniujiao | Bubali Cornu | buffalo horn |
| **天花粉** | Tianhuafen | Trichosanthis Radix | Trichosanthes root, Skegourd root |
| **菟丝子** | Tusizi | Cuscutae Semen,Semen Cuseutae; Semen Cuseutae | Dodder Seed |
| **吴茱萸** | Wuzhuyu | Euodiae Fructus | Medicil Evodia Fruit |
| **五味子** | Wuweizi | Schisandrae Chinensis Fructus | Schisandrae Chinensis Fructus |
| **西洋参** | Xiyangshen | Panacis Quinquefolii Radix | American Ginseng |
| **香薷** | Xiangru | Moslae Herba | all-grass of Haichow Elsholtzia |
| **玄参** | Xiangru | Moslae Herba | all-grass of Haichow Elsholtzia |
| **薏苡仁** | Yiyiren | Semen Coicis, Coicis Semen | seed of Jobstears |
| **银柴胡** | Yincha | Stellariae Radix | Starwort Root |
| **远志** | Yuanzhi | Polygalae Raedix | Thinleaf Milkwort Root |
| **皂角刺** | Zhaojiaoci | Gleditsiae Spina | spine of Chinese Honeylocust |
| **泽泻** | Zexie | Rhizoma Alismatis, Alismatis Rhizoma | rhizome of Oriental Waterplantain |
| **知母** | Zhimu | Rhizoma Anemarrhee, Anemarrhenae Rhizoma | rhizome of Common Amarrhe |
| **栀子** | Zhizi | Gardeniae Fructus | fruit of Cape Jasmine |
| **炙甘草** | Zhigancao | Glycyrrhizae Radix Et Rhizoma Praeparata Cum Melle | liquorice root |
| **猪苓** | Zhuling | Polyporus | Polyporus grifolia, Umbellate pore-fungus |
