# Supplementary material for: Visualization of Traditional Chinese Medicine Formulas: Development and Usability Study
Source: JMIR Form Res. 2023 Apr 21;7:e40805. doi: 10.2196/40805 (PMC10163399; doi:10.2196/40805)
Supplement: Multimedia Appendix 2 [file formative_v7i1e40805_app2.pdf]

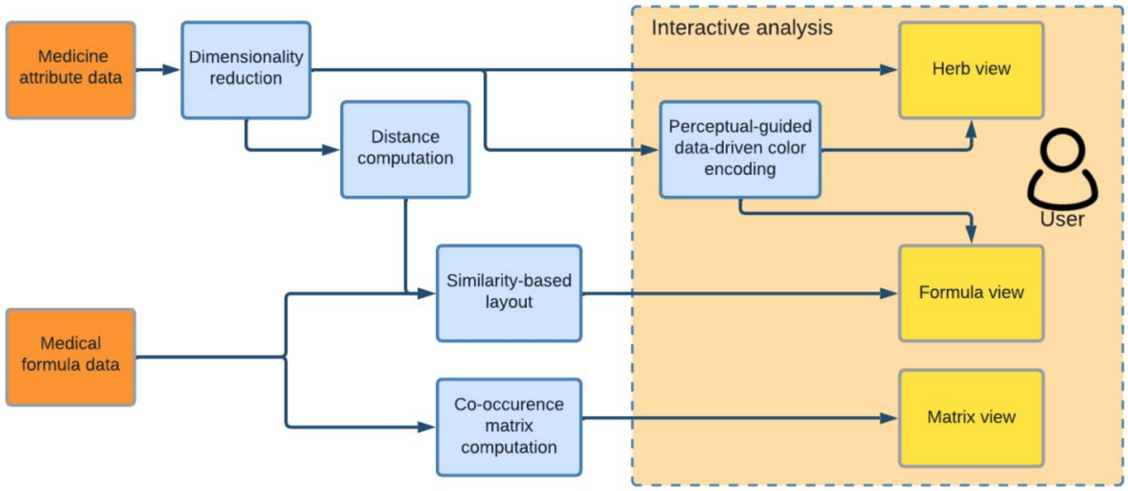
Medicine attribute data
Dimensionality reduction
Distance computation
Interactive analysis
Herb view
Perceptual-guided data-driven color encoding
User
Similarity-based layout
Formula view
Medical formula data
Co-occurence matrix computation
Matrix view

Fig 1

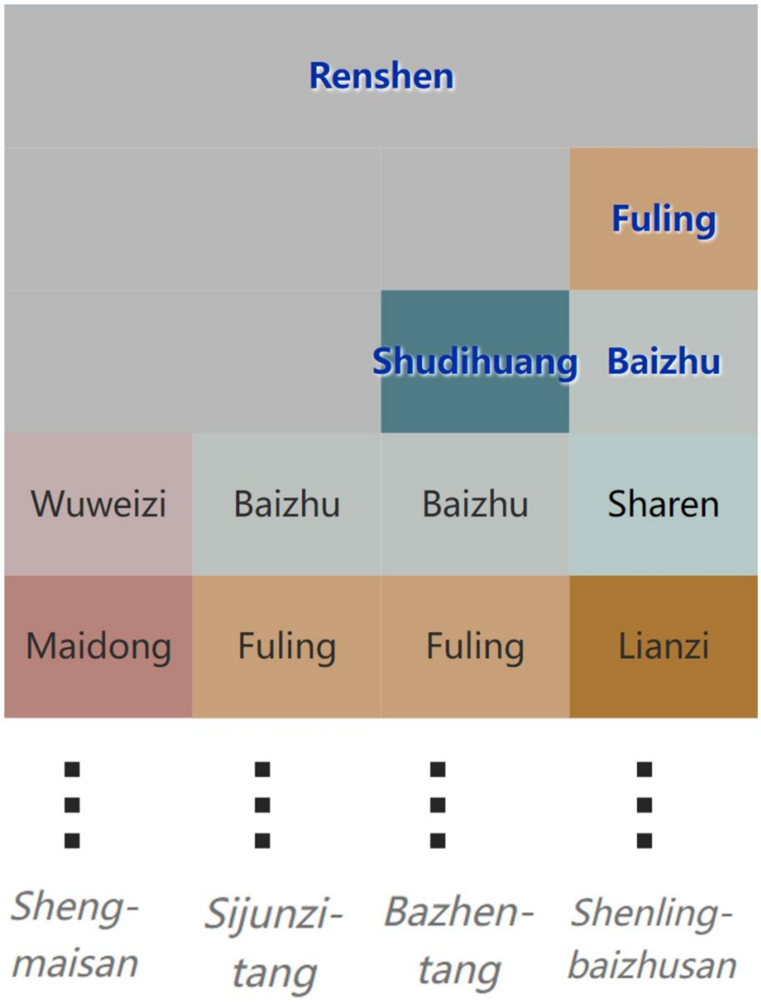
Renshen
Fuling
Shudihuang
Baizhu
Wuweizi
Baizhu
Baizhu
Sharen
Maidong
Fuling
Fuling
Lianzi
Sheng-maisan
Sijunzi-tang
Bazhen-tang
Shenling-baizhusan

Fig 2

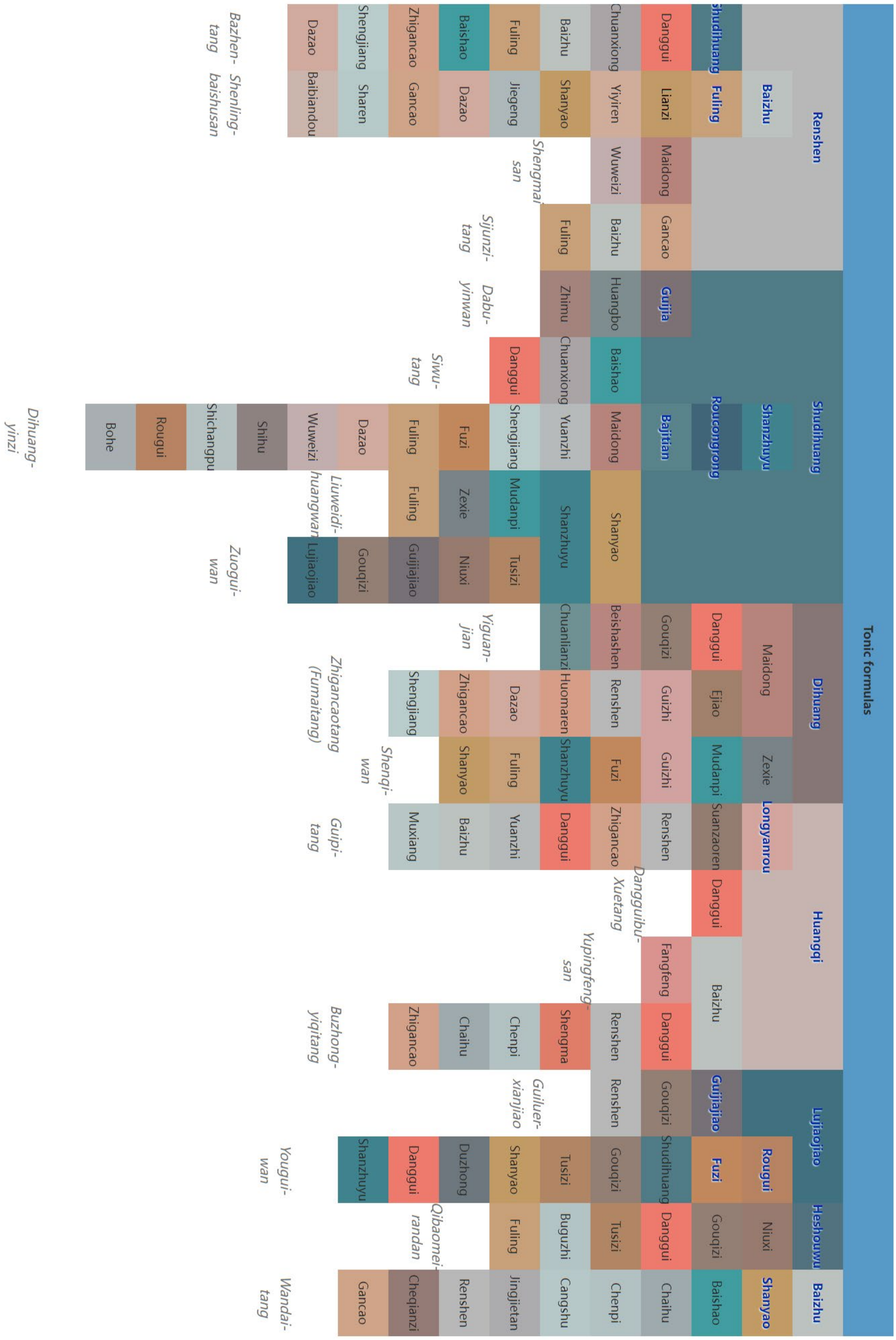
Tonic formulas
Renshen
Shudihuang
Dihuang
Huangqi
Lujiaojiao
Heshouwu
Baizhu
Bazhen-tang
Shenling-baishusan
Shengmai-san
Sijunzi-tang
Dabu-yinwan
Siwu-tang
Dihuang-yinzi
Liuweidi-huangwan
Zuogui-wan
Yiguan-jian
Zhigancaotang (Fumaitang)
Shenqi-wan
Guipi-tang
Dangguibu-Xuetang
Yupingfeng-san
Buzhong-yiqitang
Guiluer-xianjiao
Yougui-wan
Qibaomei-randan
Wandai-tang

Fig 3(A)

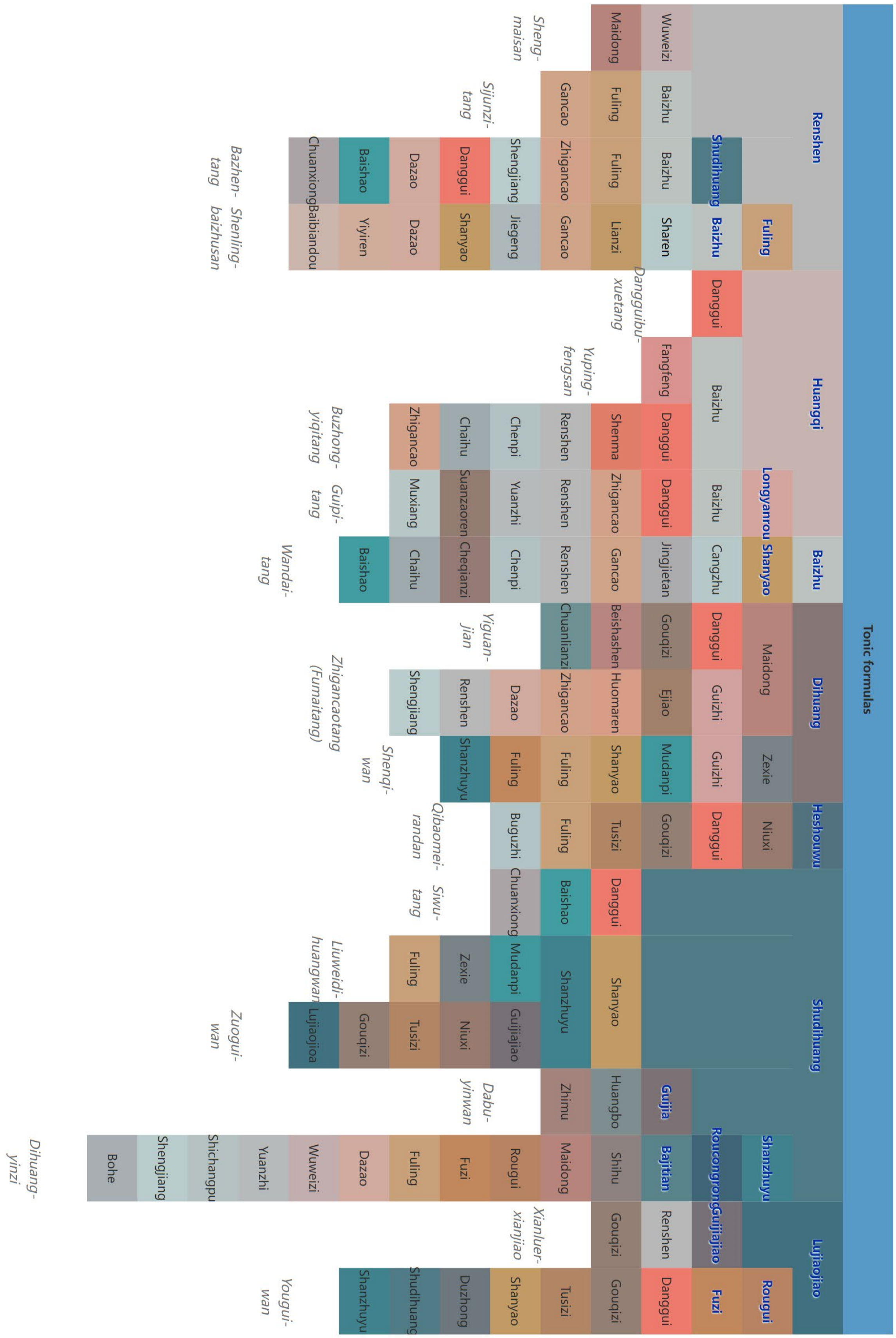
Tonic formulas
Renshen
Huangqi
Baizhu
Dihuang
Heshouwu
Shudihuang
Lujiaojiao
Shengmaisan
Sijunzitang
Bazhentang
Shenlingbaizhusan
Dangguibuxuetang
Yupingfengsan
Buzhongyiqitang
Guipitang
Wandaitang
Yiguanjian
Zhigancaotang (Fumaitang)
Shenqiwan
Qibaomeirandan
Siwutang
Liuweidihuangwan
Zuoguiwan
Dabuyinwan
Dihuangyinzi
Xianluerxianjiao
Youguiwan

Fig 3(B)

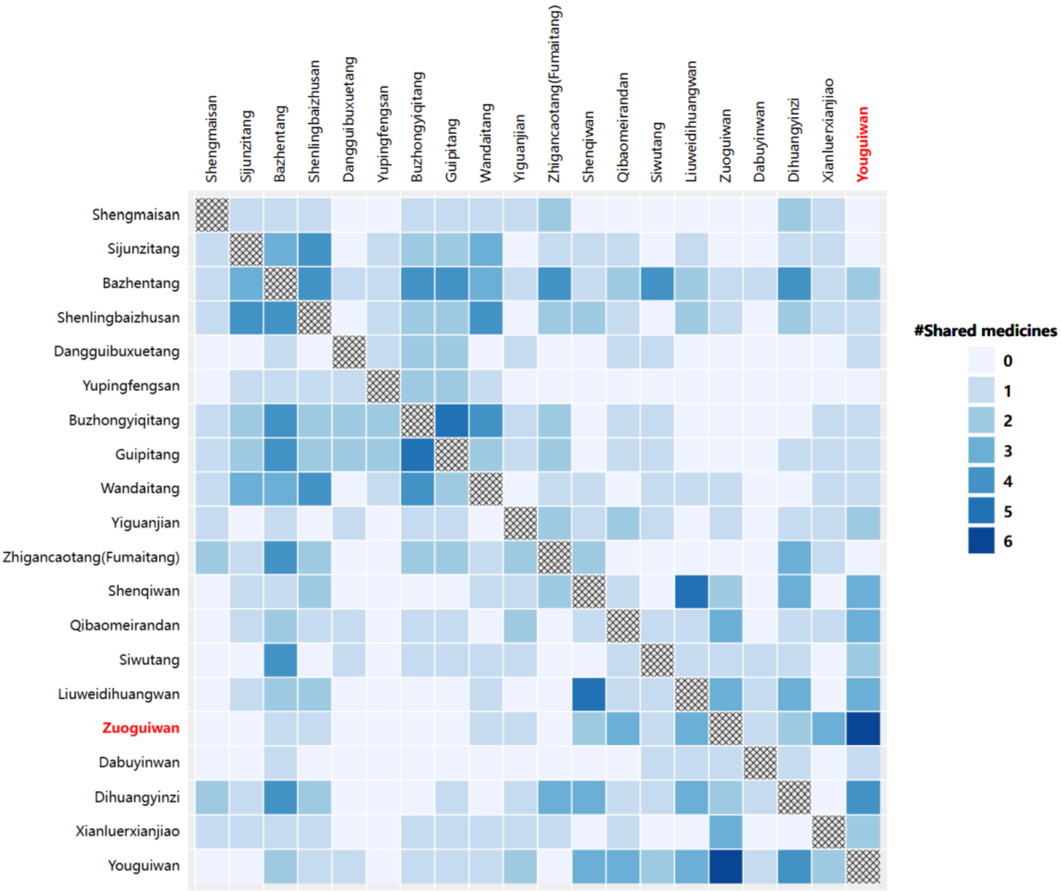
Shengmaisan
Sijunzitang
Bazhentang
Shenlingbaizhusan
Dangguibuxuetang
Yupingfengsan
Buzhongyiqitang
Guipitang
Wandaitang
Yiguanjian
Zhigancaotang(Fumaitang)
Shenqiwan
Qibaomeirandan
Siwutang
Liuweidihuangwan
Zuoguiwan
Dabuyinwan
Dihuangyinzi
Xianluerxianjiao
Youguiwan
#Shared medicines
0
1
2
3
4
5
6

Fig 4

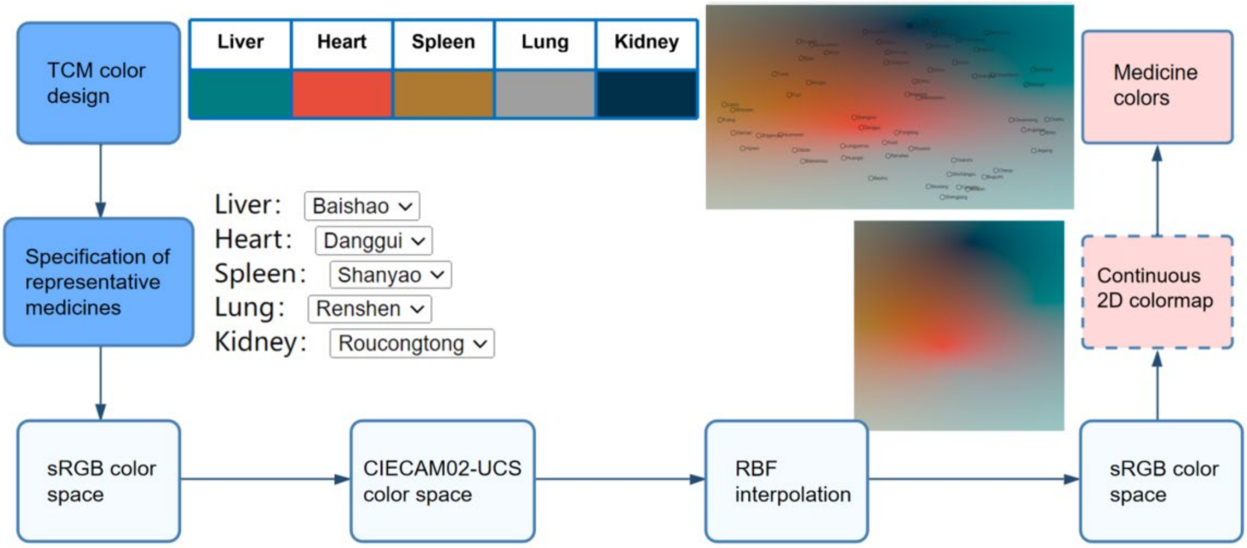
TCM color design
Liver
Heart
Spleen
Lung
Kidney
Medicine colors
Liver: Baishao
Heart: Danggui
Spleen: Shanyao
Lung: Renshen
Kidney: Roucongtong
Specification of representative medicines
Continuous 2D colormap
sRGB color space
CIECAM02-UCS color space
RBF interpolation
sRGB color space

Fig 5

Five elements	Wood	Fire	Earth	Metal	Water
Five colors	Cyan	Red	Yellow	White	Black
Five internal organs	Liver	Heart	Spleen	Lung	Kidney
Colors					

Fig 6

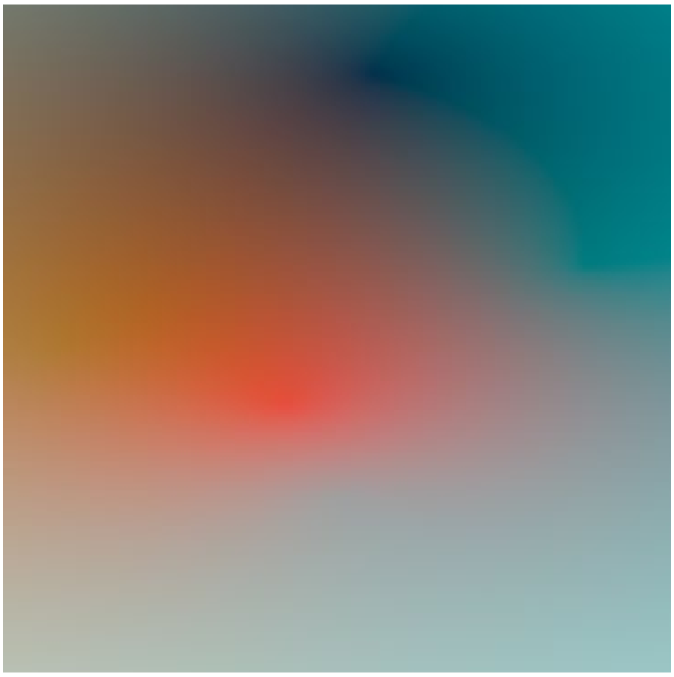

Fig 7(A)

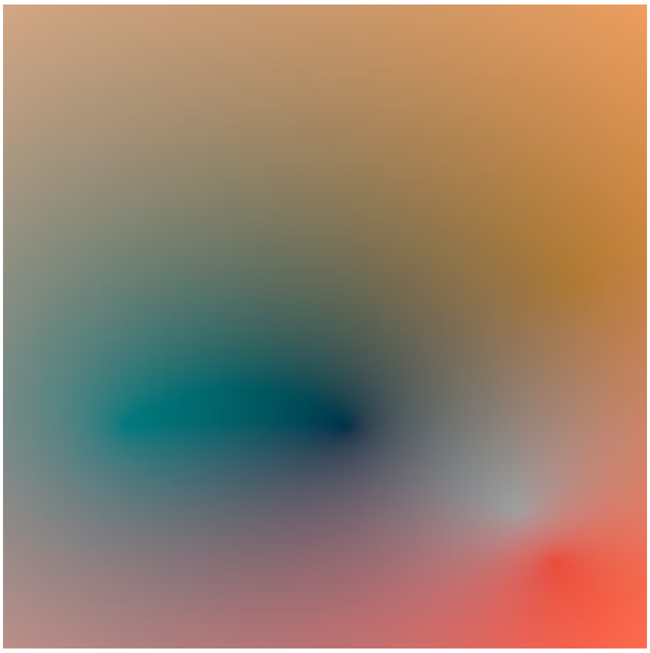

Fig 7(B)

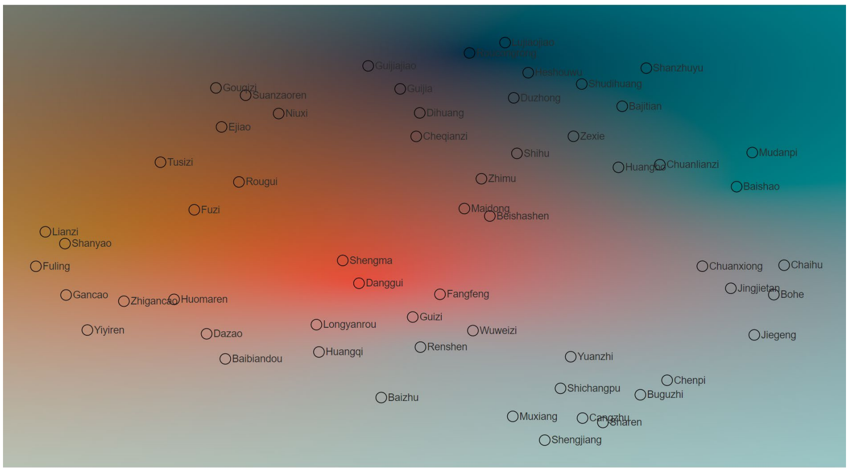

Fig 7(C)

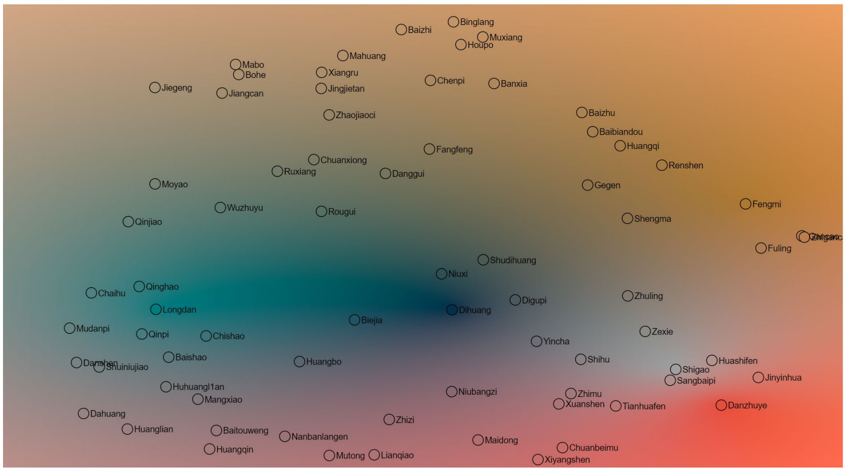

Fig 7(D)

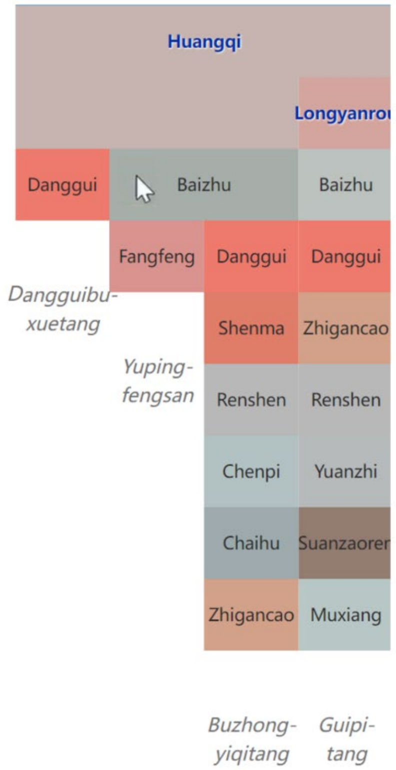

Possible formulas: Buzhongyiqitang★、Yupingfengsan★

Fig 8(A)

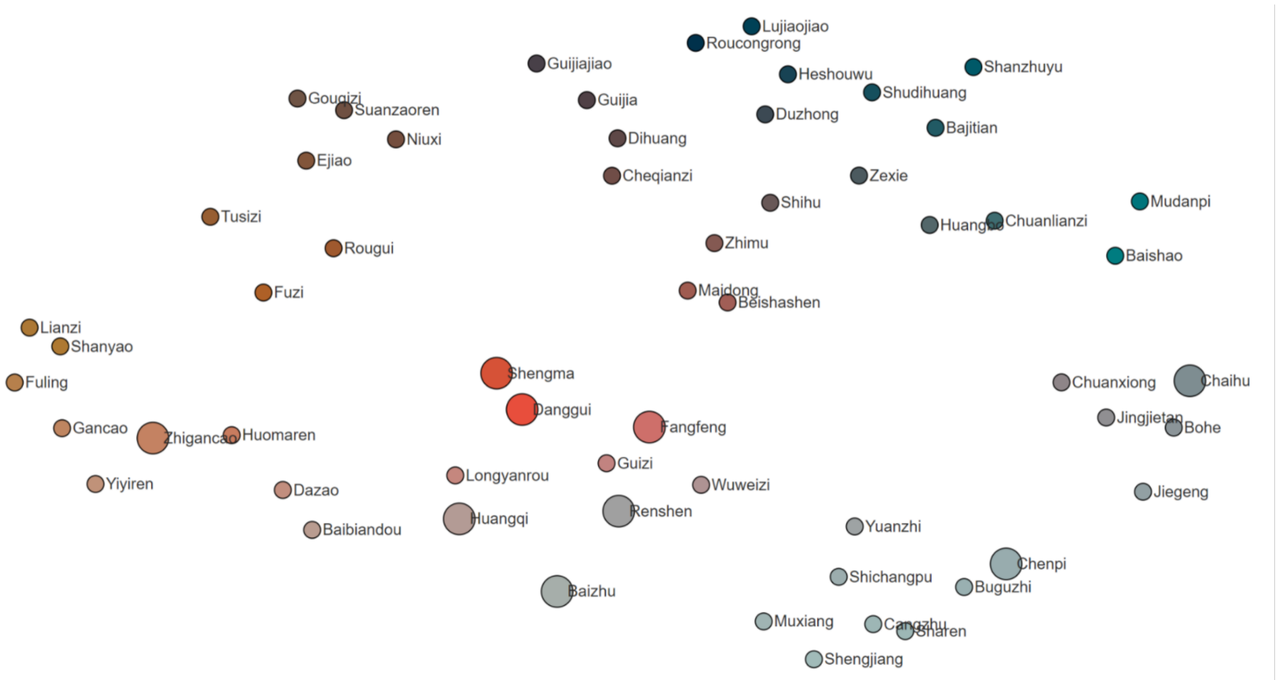

Possible formulas: Buzhongyiqitang★、Yupingfengsan★

Fig 8(B)

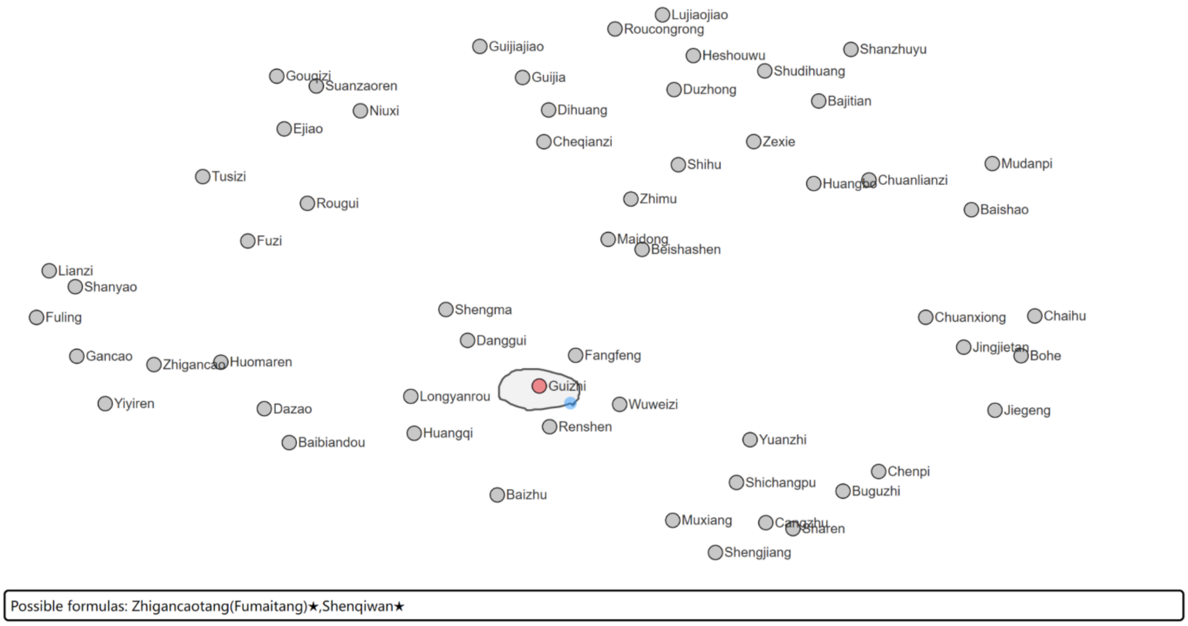
Lujiaojiao
Roucongrong
Guijiajiao
Heshouwu
Shanzhuyu
Shudihuang
Gouqizi
Suanzaoren
Guijia
Duzhong
Bajitian
Niuxi
Dihuang
Ejiao
Cheqianzi
Zexie
Shihu
Mudanpi
Tusizi
Chuanlianzi
Rougui
Zhimu
Baishao
Fuzi
Maidong
Beishashen
Lianzi
Shanyao
Shengma
Chuanxiong
Chaihu
Fuling
Danggui
Jingjie
Bohe
Fangfeng
Gancao
Zhigancao
Huomaren
Guizhi
Longyanrou
Wuweizi
Yiyiren
Dazao
Jiegeng
Renshen
Huangqi
Yuanzhi
Baibiandou
Chenpi
Shichangpu
Buguzhi
Baizhu
Muxiang
Sharen
Shengjiang
Possible formulas: Zhigancaotang(Fumaitang)★,Shenqiwan★

Fig 8(C)

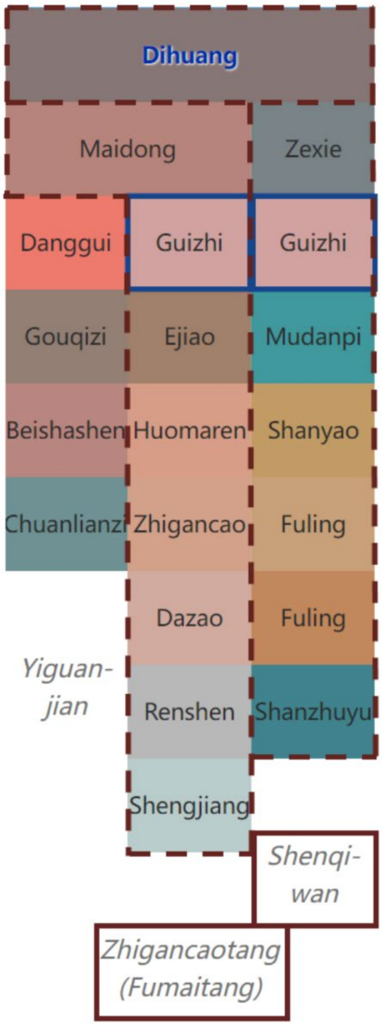
Dihuang
Maidong
Zexie
Danggui
Guizhi
Guizhi
Gouqizi
Ejiao
Mudanpi
Beishashen
Huomaren
Shanyao
Chuanlianzi
Zhigancao
Fuling
Dazao
Fuling
Yiguan-jian
Renshen
Shanzhuyu
Shengjiang
Shenqi-wan
Zhigancaotang (Fumaitang)

Fig 8(D)

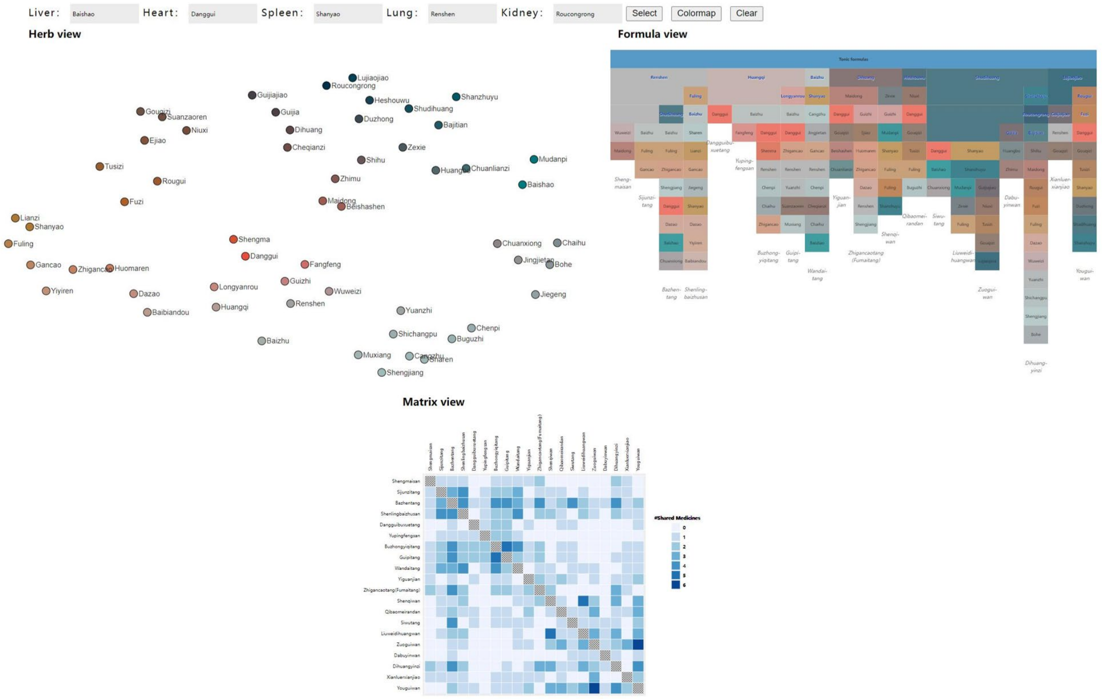

(A) Tonic formulas.

Fig 9(A)

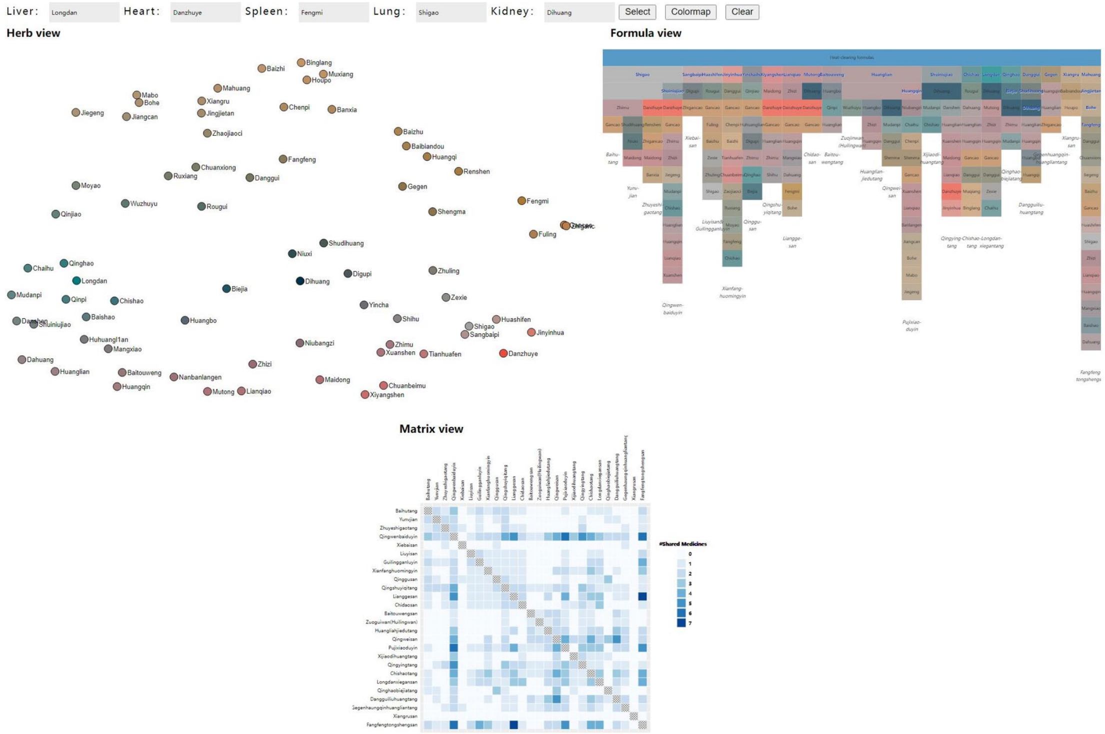

(B) Heat-clearing formulas.

Fig 9(B)

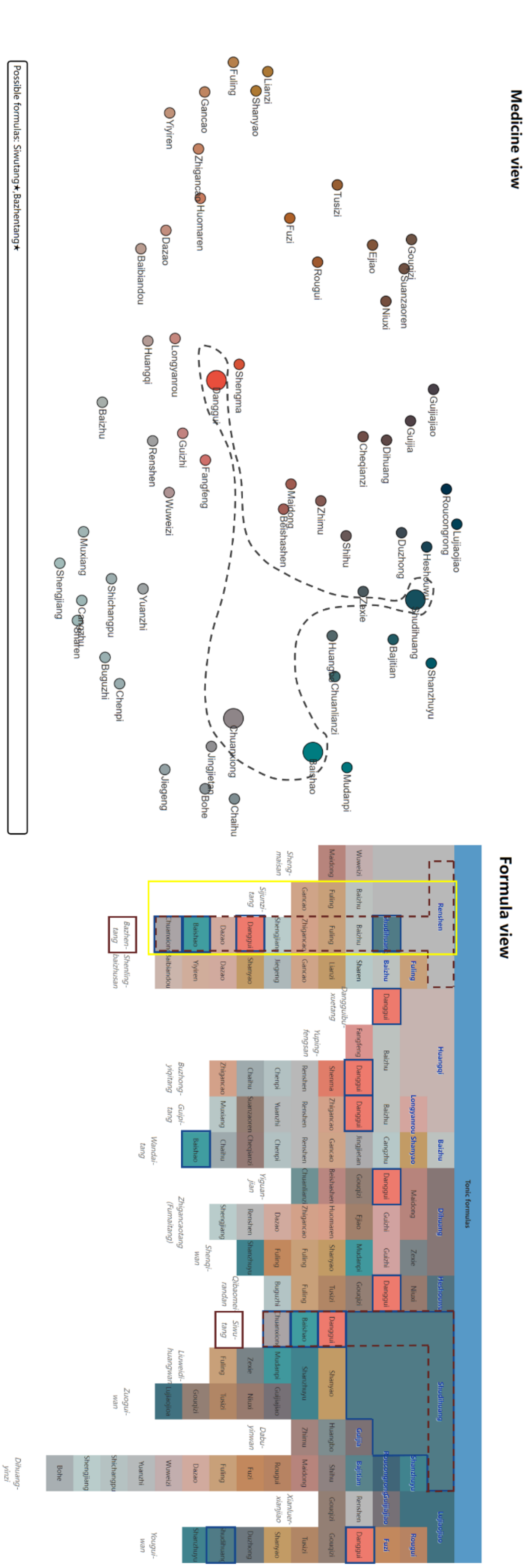
Medicine view
Formula view
Possible formulas: Siwutang★,Bazhentang★
Tonic formulas

Fig 10

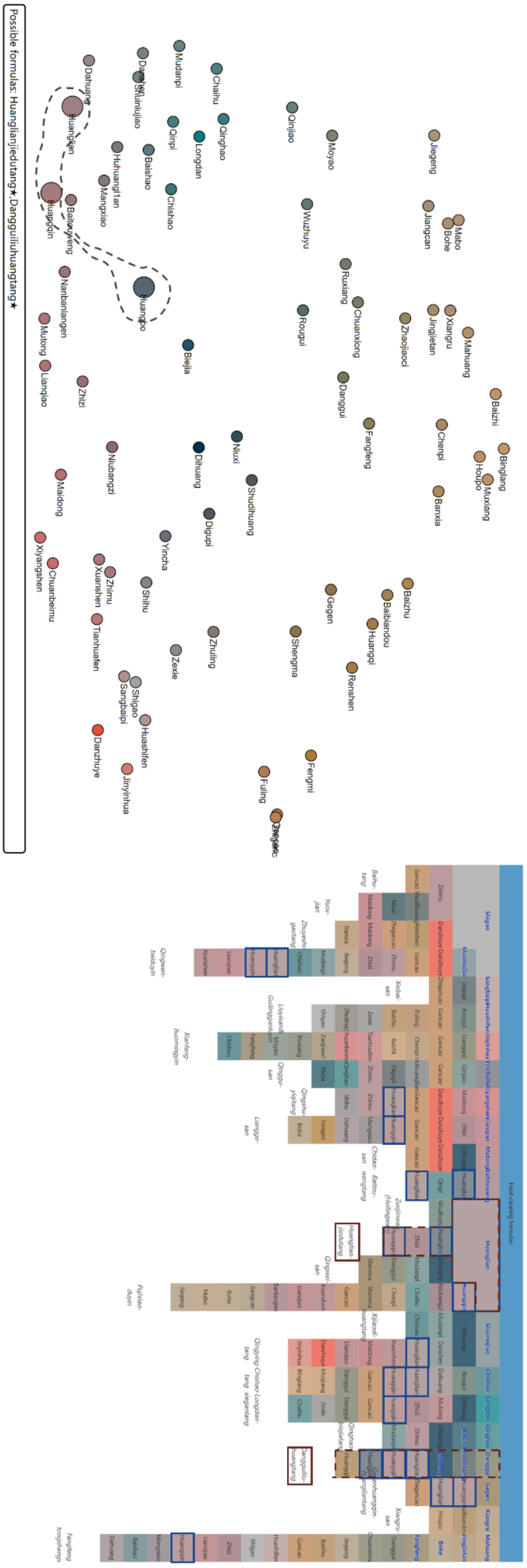

Medicine view
Formula view
Possible formulas: Huanglianjiedutang★,Dangguiliuhuangtang★

Fig 11
