# Supplementary figures and images for: Visualization of Traditional Chinese Medicine Formulas: Development and Usability Study
Source: JMIR Form Res. 2023 Apr 21;7:e40805. doi: 10.2196/40805 (PMC10163399; doi:10.2196/40805)

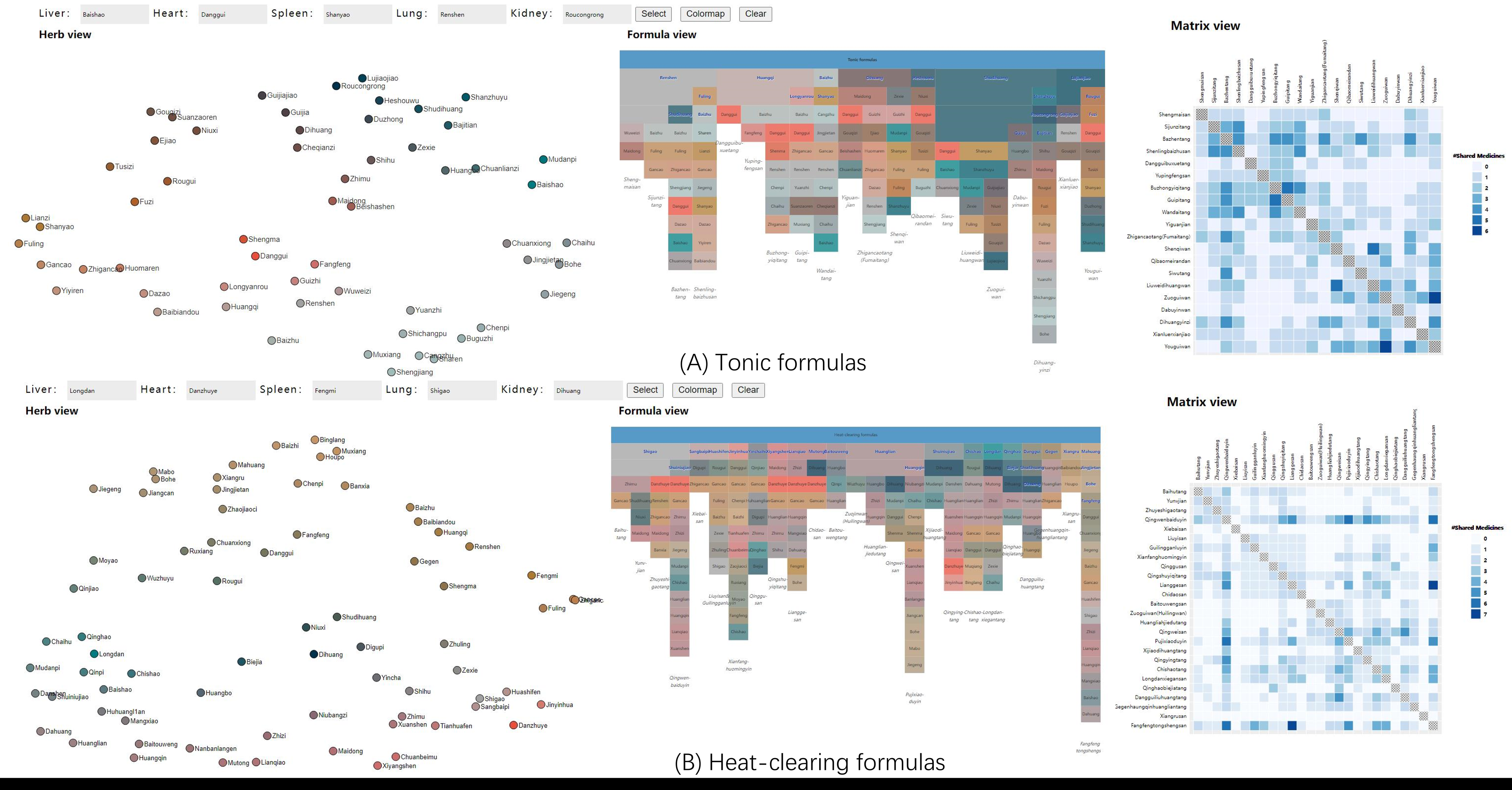

Supplement: Multimedia Appendix 3 [file formative_v7i1e40805_app3.png]

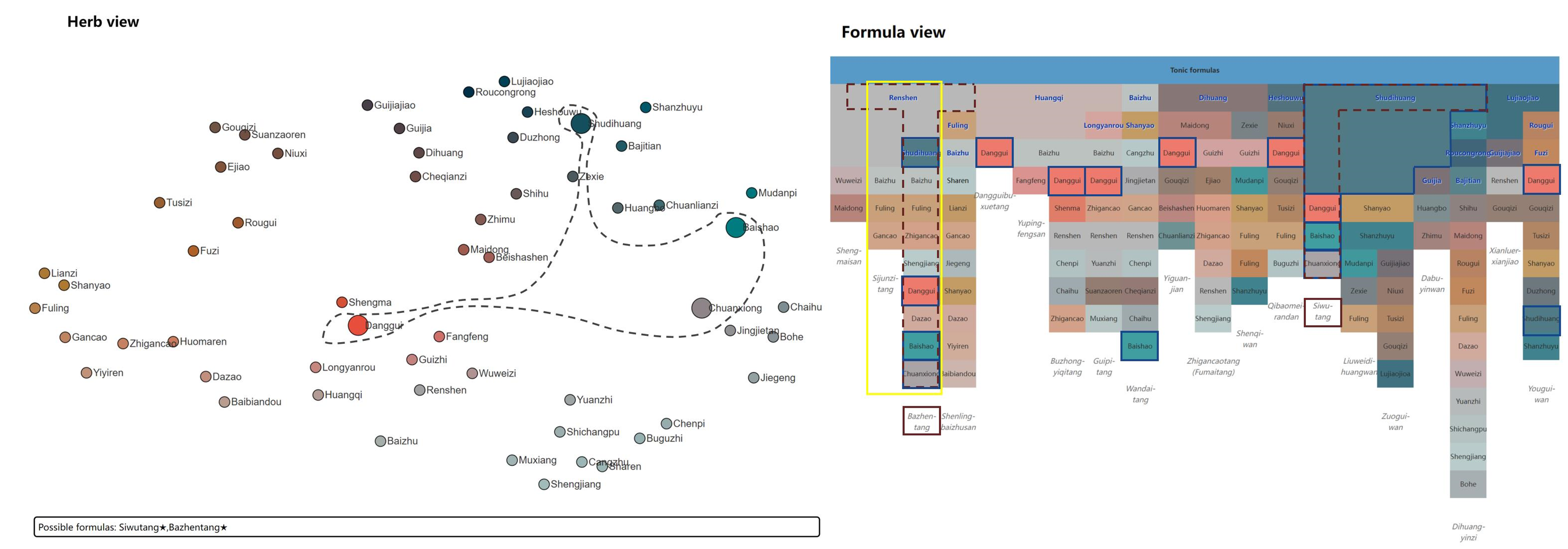

Supplement: Multimedia Appendix 4 [file formative_v7i1e40805_app4.png]

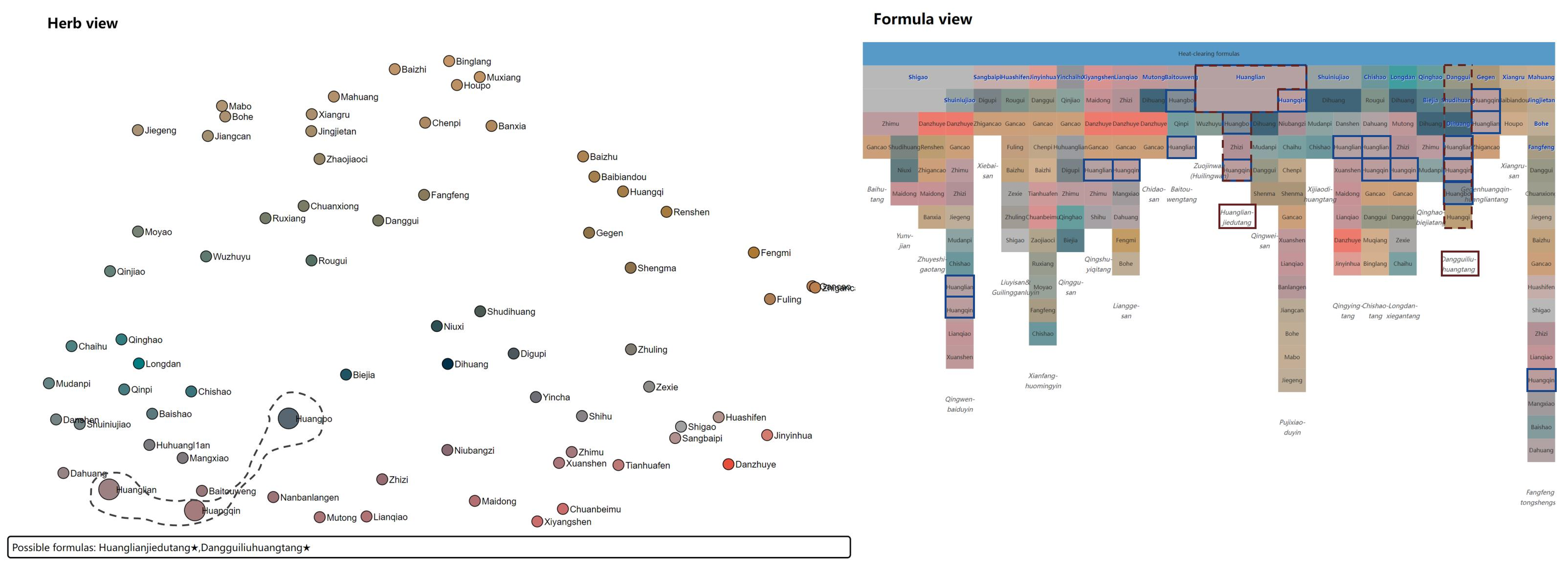

Supplement: Multimedia Appendix 5 [file formative_v7i1e40805_app5.png]
